# DNA methylation clocks tick in naked mole rats but queens age more slowly than nonbreeders

**DOI:** 10.1038/s43587-021-00152-1

**Published:** 2021-12-23

**Authors:** Steve Horvath, Amin Haghani, Nicholas Macoretta, Julia Ablaeva, Joseph A. Zoller, Caesar Z. Li, Joshua Zhang, Masaki Takasugi, Yang Zhao, Elena Rydkina, Zhihui Zhang, Stephan Emmrich, Ken Raj, Andrei Seluanov, Chris G. Faulkes, Vera Gorbunova

**Affiliations:** 1 Department of Human Genetics, David Geffen School of Medicine, University of California, Los Angeles, Los Angeles, CA, USA.; 2 Department of Biostatistics, Fielding School of Public Health, University of California, Los Angeles, Los Angeles, CA, USA.; 3 Altos Labs, San Diego, CA, USA.; 4 Departments of Biology and Medicine, University of Rochester, Rochester, NY, USA.; 5 Radiation Effects Department, Centre for Radiation, Chemical and Environmental Hazards, Public Health England, Chilton, Didcot, UK.; 6 School of Biological and Chemical Sciences, Queen Mary University of London, London, UK.; 7 These authors contributed equally: Steve Horvath, Amin Haghani.; 8 These authors jointly supervised this work: Andrei Seluanov, Chris G. Faulkes, Vera Gorbunova.

## Abstract

Naked mole rats (NMRs) live an exceptionally long life, appear not to exhibit age-related decline in physiological capacity and are resistant to age-related diseases. However, it has been unknown whether NMRs also evade aging according to a primary hallmark of aging: epigenetic changes. To address this question, we profiled *n* = 385 samples from 11 tissue types at loci that are highly conserved between mammalian species using a custom array (HorvathMammalMethylChip40). We observed strong epigenetic aging effects and developed seven highly accurate epigenetic clocks for several tissues (pan-tissue, blood, kidney, liver, skin clocks) and two dual-species (human–NMR) clocks. The skin clock correctly estimated induced pluripotent stem cells derived from NMR fibroblasts to be of prenatal age. The NMR epigenetic clocks revealed that breeding NMR queens age more slowly than nonbreeders, a feature that is also observed in some eusocial insects. Our results show that despite a phenotype of negligible senescence, the NMR ages epigenetically.

The NMR, *Heterocephalus glaber*, has emerged as a popular model for aging studies due to its exceptional maximum lifespan (37 yr), despite being of comparable size to a laboratory mouse, with a maximum lifespan of 4 yr. NMRs are found in the arid regions of East Africa, living in colonies that have a eusocial structure^1^, similar to those of ants, bees and wasps. NMR social groups consist of a single breeding queen who mates with one to three specific breeding males, while the rest of the colony are non-reproductive and most will never breed. This hystricomorph rodent appears to be resistant to many age-related diseases^2-8^, including two leading causes of death in humans: cardiovascular disease and cancer^9,10^. Strikingly, the mortality rate of this rodent defies the Gompertz–Makeham law by not increasing with age^11^. The NMR is proposed to be a ‘nonaging’ mammal as it displays negligible senescence, including minimal age-related decline in physiological capacity as measured by several aging biomarkers^3,11^.

Cytosine methylation is one of the best characterized epigenetic modifications that modulates gene activity and chromatin structure. In mammals, DNA methylation plays an important role in multiple biological processes including silencing of transposable elements, regulation of gene expression, genomic imprinting, X-chromosome inactivation, carcinogenesis and aging^12^. Indeed, it was long observed that the degree of cellular DNA methylation changes with age in humans and many mammalian species^13-15^. Methylation levels of multiple CpGs can be consolidated to develop highly accurate multivariate age-estimators (epigenetic clocks) for all human tissues^16-18^. Epigenetic age, as measured by an epigenetic clock, is arguably the most accurate estimator of age in numerous mammalian species including humans^17,19-21^. Hence, it remains to be ascertained whether the NMR epigenome undergoes age-related changes. The human epigenetic clocks, however, cannot be applied to nonprimate species because of evolutionary genome sequence divergence^16^. Hence, it is necessary to develop de novo epigenetic clocks that are specific to NMRs.

A previous study used methylation data at 51 CpGs to build a preliminary epigenetic age estimator in *n* = 24 NMR liver samples^22^. However, the sample size and the selection of tissues used in this previous study was insufficient for building a robust clock that could lend itself for gaining biological insights. Here we used another measurement platform (mammalian methylation array) to generate a large dataset from 11 NMR tissues and cell types. We present epigenetic clocks for the NMR and demonstrate that despite a phenotype of reduced senescence, NMRs, similar to other mammals, age at the epigenetic level in all of the tissues we examined. We also reveal the characteristics of individual methylated loci that correlate strongly with age in different NMR tissues. We present evidence that NMR queens exhibit slower epigenetic aging rates and characterize CpGs that relate to queen status.

## Results

### Random forest predictors of tissue and sex.

**Extended Data Fig. 1 ∣:**
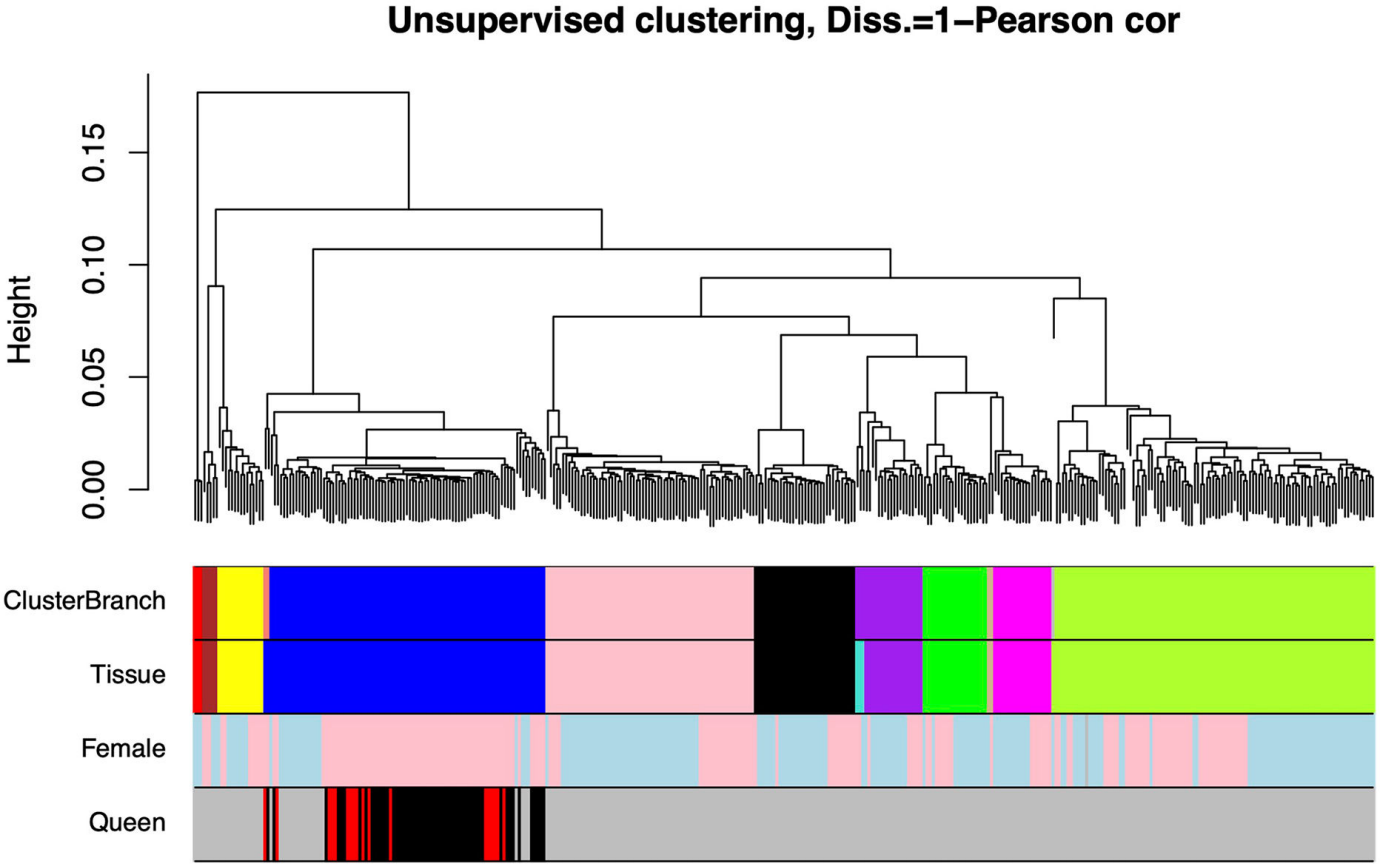
Unsupervised hierarchical clustering of NMR tissues. Average linkage hierarchical clustering based on the inter-array correlation coefficient (Pearson correlation). The first color band corresponds to branches/clusters defined at a height value of 0.04 (y-axis). The second color band encodes tissue type: adipose (turquoise, n = 3), blood (blue, n = 92), cerebellum (brown, n = 5), cortex (yellow, n = 15), heart (green, n = 21), iPSC (red, n = 3), kidney (black, n = 33), liver (pink, n = 68), lung (magenta, n = 19), muscle (purple, n = 19), skin (greenyellow, n = 105), spleen (tan, n = 2). The third color band encodes sex (pink=female, lightblue=male). The fourth color band encodes queen status in females (red=queen, black=non-breeding female, grey=male samples or females with unknown reproductive status). Contrasting the first color band (based on cluster branches) with the second color band shows that the samples cluster by tissue type.

We obtained methylation profiles of 382 DNA samples derived from 11 NMR tissues (Supplementary Table 1): adipose (*n* = 3), blood (*n* = 92), cerebellum (*n* = 5), cerebral cortex (*n* = 15), heart (*n* = 21), kidney (*n* = 33), liver (*n* = 68), lung (*n* = 19), muscle (*n* = 19), skin (*n* = 105) and spleen (*n* = 2), and induced pluripotent stem cells. These tissues were obtained from NMRs that ranged in age from 0 to 26 yr. All methylation profiles were generated using the mammalian methylation array (HorvathMammalMethylChip40), in which 27,917 of its 37,492 CpGs mapped to the NMR genome (HetGla_female_1.0.100 genome assembly). Random forest predictors for tissue type and sex based on these methylation profiles led to near-perfect accuracy in predicting tissue type (out-of-bag error rate of zero) and sex (only 1 misclassification from 385 samples) (Extended Data Fig. 1). The remarkable accuracy of these predictors based on methylation profiles makes them valuable to validate platemaps and to detect potential human errors that may occur when deriving DNA methylation data.

### Epigenetic clocks.

**Fig. 1 ∣:**
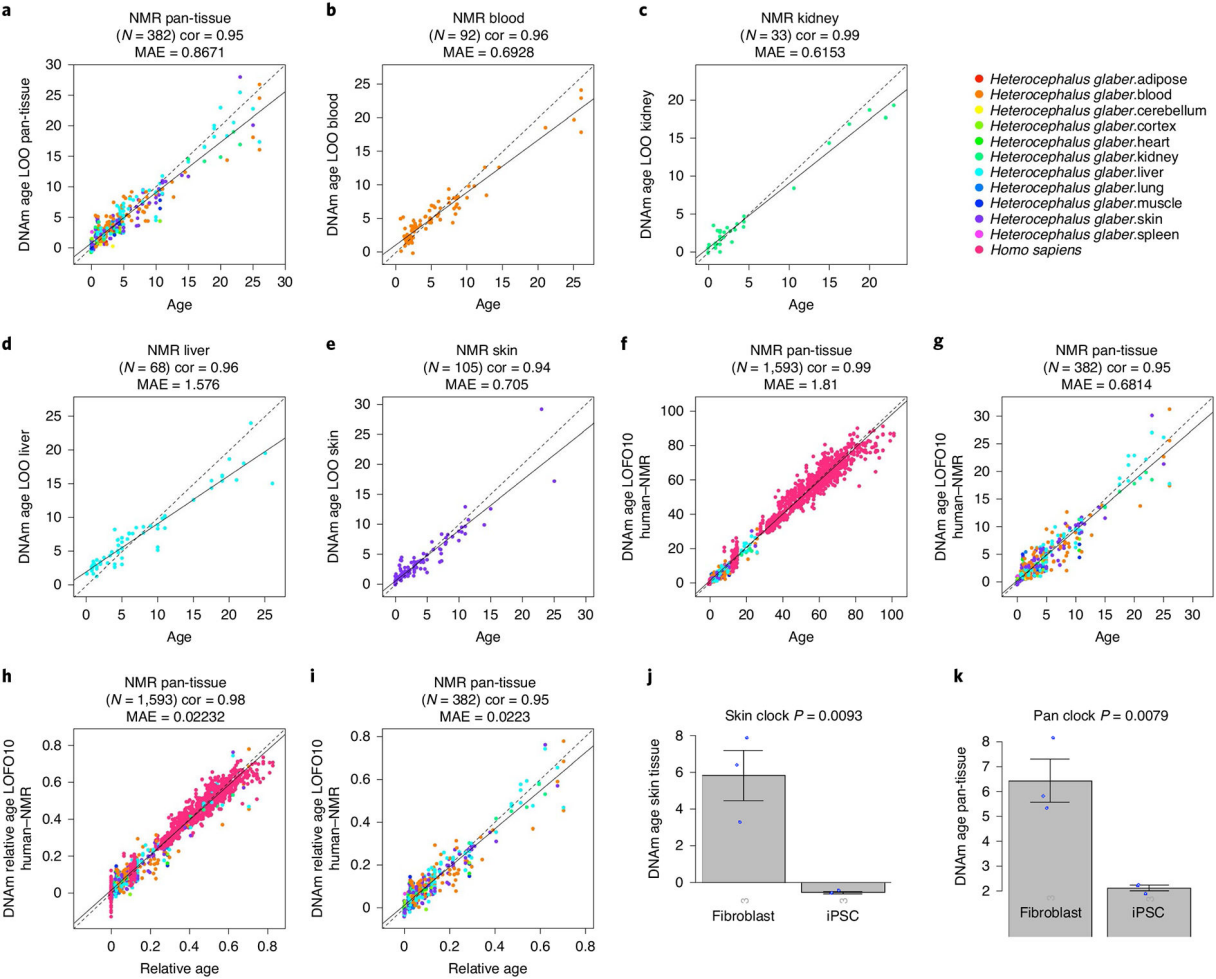
Epigenetic clocks for NMRs and humans. **a**–**i**, Chronological age versus cross-validation estimates of DNA methylation age (*y* axis, in units of years) for different epigenetic clocks: pan-tissue epigenetic clock (**a**), blood clock (**b**), kidney clock (**c**), liver clock (**d**), skin clock (**e**), human–NMR clock for chronological age (**f**, **g**), human–NMR clock for relative age (**h**, **i**). We used two validation schemes: leave-one-out cross-validation (LOO) (**a**–**e**) and tenfold cross-validation balanced for species (LOFO10) (**f**–**i**). **f**,**g**, Human–NMR clock for chronological age applied to both species (**f**) and NMR only (**g**). **h**,**i**, Human–NMR clock for relative age applied to both species (**h**) and NMR only (**i**). Dots are colored by species (red, human) (**f**, **h**) or NMR tissue type (**g**, **i**). **h**,**i**, Excerpts of panels **f** and **h**, respectively, but restricted to NMR samples. Each panel reports the sample size, Pearson correlation coefficient, median absolute error (MAE). **j**,**k**, Independent test set analysis of iPS cells and fibroblasts from NMR using the NMR skin clock (**j**) and the NMR pan-tissue clock (**k**). The bar plots report the mean values, 1 s.e.m., two-sided Student’s *t*-test and the group sizes (*n* = 3 fibroblasts and *n* = 3 iPSC cells). cor, Pearson correlation coefficient; LOFO10, 10 fold cross validation, leave-one-fraction-out.

To build methylation-based age-estimators, we used penalized regression models (elastic net regression) to regress chronological age (dependent variable) on the 27,917 CpGs (covariates) that map to the NMR genome. To arrive at unbiased estimates of the epigenetic clocks, we performed cross-validation analyses and obtained estimates of age correlation *R* (Pearson correlation between estimated DNA methylation (DNAm) age and chronological age), as well as the median absolute error. We generated four epigenetic clocks for NMRs that are optimized for blood, kidney, liver and skin, respectively. In addition, we also developed a fifth epigenetic clock that is applicable to all NMR tissues: the NMR pan-tissue clock (Fig. 1). None of these clocks used induced pluripotent stem cells (iPSC) in their construction.

**Extended Data Fig. 2 ∣:**
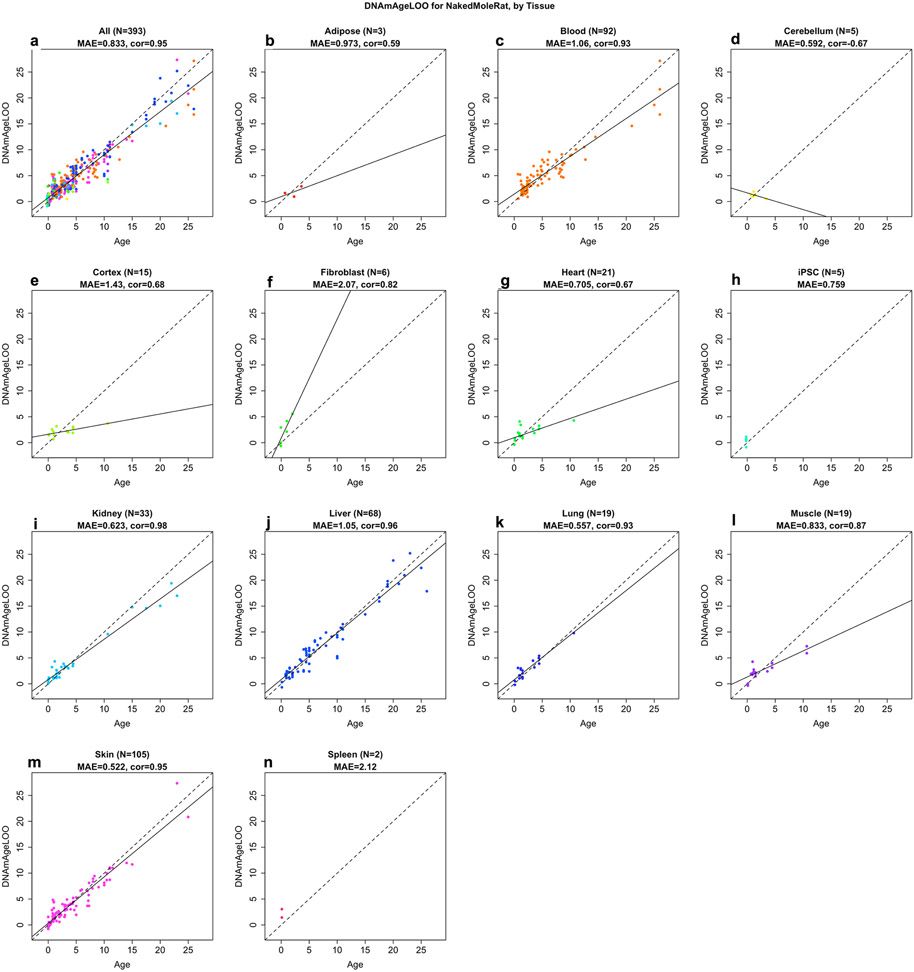
Pan tissue clock for naked mole-rat applied to individual tissues. **a**, All tissues combined. Points correspond to tissue samples that are color coded as indicated in the remaining panels **b**, Adipose (red color), **c**, Blood (darkorange color), **d**, Cerebellum (greenyellow color), **e**, Cerebral cortex (lightgreen), **f**, Heart (green), **g**, Kidney, **h**, Liver (lightblue), **i**, Lung (blue), **j**, Muscle (purple), **k**, Skin (magenta), **l**, Spleen. Each panel depicts the leave-one-out estimate of age (y-axis) versus chronological age (x-axis). Both axes report values in units of years. Each title reports the sample size (N), Pearson correlation coefficient, and median absolute error. The solid line results from a linear regression model. Note that panels **b,d,l** involve very few samples with a limited age range.

Pan-tissue, blood, kidney, liver and skin clocks generated very strong age correlations (*R* ≥ 0.94, Fig. 1a-e). A detailed analysis of the performance of the pan-tissue clock with different tissues is presented in Extended Data Fig. 2. Tissues with large numbers of samples (blood, kidney, liver and skin) showed excellent correlation between DNAm age and chronological age. Overall, these NMR clocks are highly accurate estimators of chronological age that the research community can readily employ in their investigations into aging.

As the aim of studying NMR aging is to ultimately understand human aging, it would be beneficial if age measurements of NMRs could be readily translated to human age. Towards this end, we developed two dual-species clocks (human–NMR). These clocks were derived by employing the elastic net regression approach as described above, but on the training dataset that consisted of DNA methylation profiles of both human and NMR tissues, which were all profiled with the mammalian methylation array. The output of one of the human–NMR clocks reports age in years, while the other reports relative age, which is the ratio of chronological age of an individual to the maximum recorded lifespan of its species, and assumes values between 0 and 1. The resulting human–NMR clock for chronological age (in years) was highly accurate, whether both species were analyzed together (*R* = 0.99, Fig. 1f) or whether the analysis was restricted to NMR (*R* = 0.95, Fig. 1g). Equally strong age correlations were obtained with the human–NMR clock for relative age, regardless of whether the analysis was carried out with both species (*R* = 0.98, Fig. 1h) or only NMRs (*R* = 0.95, Fig. 1i). These results suggest that a subset of CpGs in both humans and NMRs undergo similar methylation changes during aging, allowing the derivation of highly accurate dual-species age-estimators. These clocks will be useful for readily translating discoveries made in NMRs to humans. The successful generation of these epigenetic clocks implies that NMRs, despite being deemed as ‘nonaging’, undergo epigenetic aging, just like other members of the mammalian class.

### iPS reprogramming reduces epigenetic age of NMR cells.

Epigenetic clocks indicate that induced pluripotent stem cells derived from adult human cells have prenatal age (that is, fetal age)^16^. To test whether the same holds true for the NMRs, we analyzed *n* = 3 iPS cell lines derived from dermal fibroblasts of NMRs^23^ and their corresponding fibroblasts from animals aged between 1 and 3 yr. Despite the low sample size (three iPS cells and three fibroblasts), all NMR clocks indicate significant rejuvenation (Student’s *t*-test *P* = 0.0093 and *P* = 0.0079, Fig. 1j,k). The skin clock indicates that iPS cells have a negative age (mean = −0.50 yr, Fig. 1j), that is, a fetal age. The mean methylation level of iPS cells is significantly lower than that of the corresponding fibroblasts (Student’s *t*-test *P* = 0.00013, iPSCs of induced pluripotent stem cells = 0.53, mean methylation of fibroblasts = 0.57). While future studies should revisit this analysis for older NMRs, we have previously found that iPSCs derived from older humans also exhibit negative epigenetic age^16^.

### Characteristics of age-related CpGs.

**Extended Data Fig. 3 ∣:**
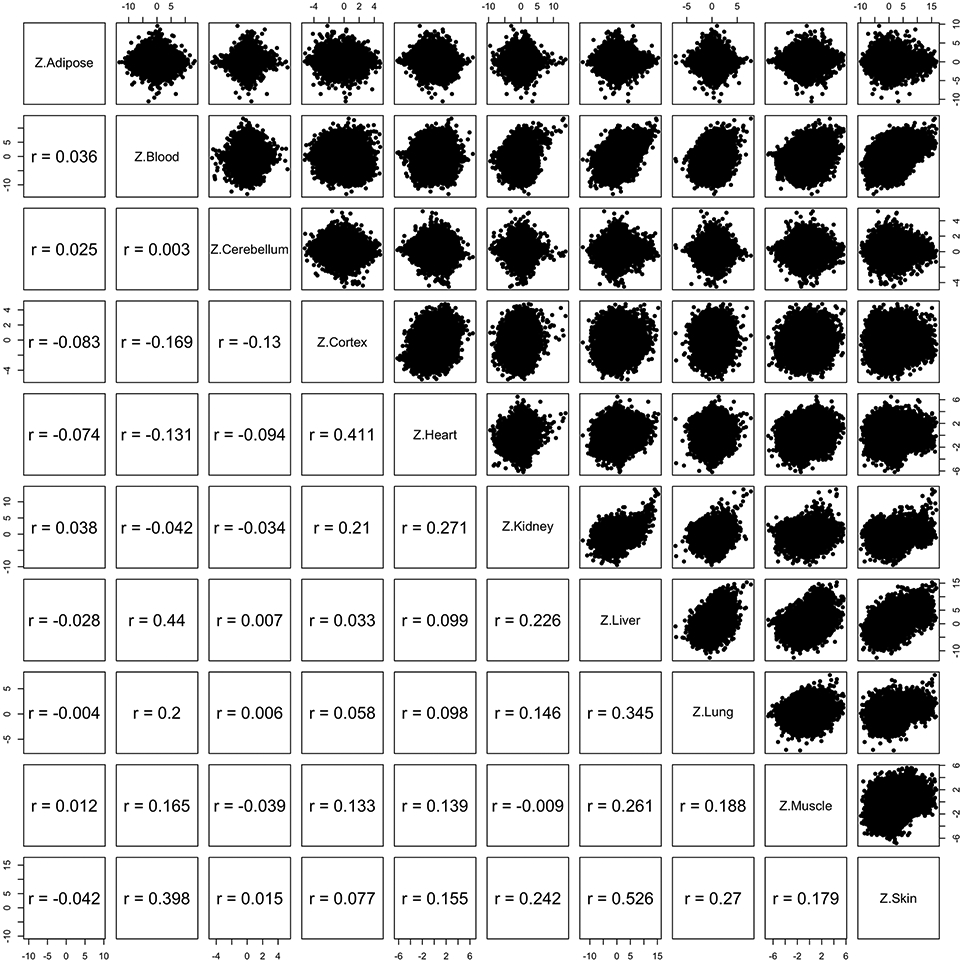
Correlation of EWAS of age in different NMR tissues. Each dot corresponds to a CpG. Student T test statistics (denoted as Z statistics) resulting from a correlation test of age in the respective tissues. The lower panels report Pearson correlation coefficients for the corresponding scatter plots. For example, the Pearson correlation between age effects in adipose (Z.Adipose) and those in the cerebellum (Z.Cerebellum) is r = 0.025.

**Fig. 2 ∣:**
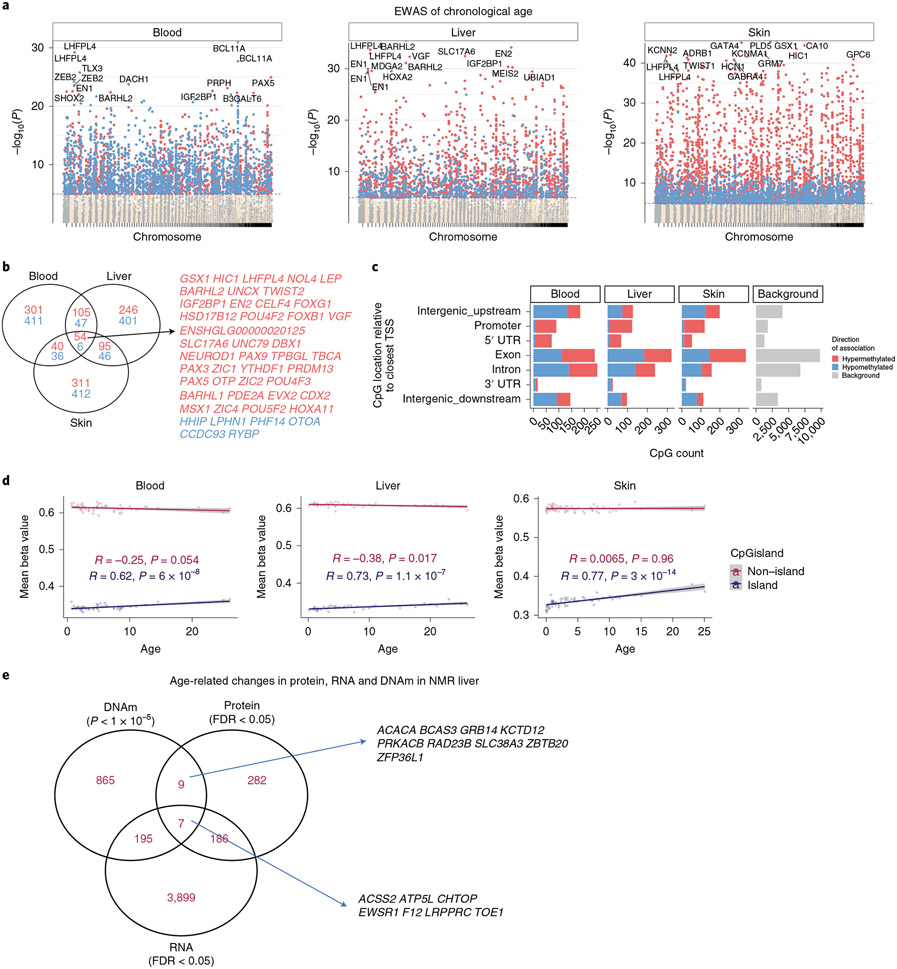
EWAS of chronological age in NMR tissues. EWAS of age in blood (*n* = 92), liver (*n* = 68) and skin (*n* = 105) of NMRs. The results from tissues with small sample sizes (cerebral cortex, heart, kidney, lung, muscle) are in Extended Data Fig. 4. **a**, Manhattan plots of the EWAS of chronological age. The genome coordinates (*x* axis) result from aligning the mammalian array probes to the HetGla_female_1.0.100 genome assembly. The directions of associations with *P* < 10^−5^ (red dotted line) are color-coded in red (age-related increase of methylation) and blue (age-related decrease in methylation). The top 15 most significant CpGs were labeled by the neighboring genes. The EWAS of age used a Pearson correlation test. The *y* axis of the Manhattan plot reports the log_10_-transformed Student’s *t*-test *P* values in each tissue (blood, liver, skin). **b**, Venn diagram of the overlap in up to the top 1,000 (500 in each direction) significant CpGs in each tissue. **c**, Location of top CpGs in each tissue relative to the closest transcriptional start site. The gray color in the last panel reports the results for the 27,917 CpGs on the mammalian array that can be mapped to the HetGla_female_1.0.100 genome. **d**, Scatter plot of mean methylation levels (mean beta values, *y* axis) versus chronological age (*x* axis) in different tissues (blood, liver, skin). The blue and red dots (and corresponding linear regression lines) correspond to mean values in CpG islands and outside of islands, respectively. Each dot is a tissue sample. *R*, Pearson correlation coefficient; *P*, correlation test *P* value. **e**, Venn diagram of genes that correlate significantly with age in liver samples from the NMR according to transcriptomic data (FDR < 0.05), proteomic data (FDR < 0.05) and DNA methylation data (nominal significance level *P* < 10^−5^). For the transcriptomic and proteomic datasets, we used FDR values provided by the authors of ref. ^18^. Details are presented in Supplementary Table 2. TSS, transcription start site; UTR, untranslated region.

To uncover epigenetic features of the NMR genome that are associated with age, we first examined the location of age-related CpGs in the genome. These were found to be distributed across all genomic structures, with some CpGs increasing and others decreasing methylation with age (Fig. 2c). Importantly, an overwhelming proportion of age-related CpGs that are located in promoters and 5′ untranslated regions (5′UTR) become increasingly methylated with age. This parallels the observed age-related gain of methylation in CpG islands (Fig. 2d). As CpG islands control the activities of many promoters, their methylation suggests the potential participation of their corresponding proximal genes in the process of aging. It is acknowledged that methylation of nonisland CpGs can also influence gene expression. To identify CpGs with methylation changes that are most related to aging, we performed an epigenome-wide association study (EWAS), where we correlated age with each of the 27,917 CpGs on the mammalian methylation array that mapped to specific loci in the *Heterocephalus_glaber_female.HefGla_female_1.0.100* genome assembly. In total, these CpGs are proximal to 4,634 genes in the NMR genome. Aging effects in one NMR tissue tend to be weakly correlated with those in another tissue (Extended Data Fig. 3), but relatively high Pearson correlations (*r* = 0.40) could be observed between skin and blood (*r* = 0.40), between skin and liver (*r* = 0.53), and between blood and liver (*r* = 0.44). This poor conservation and the differences in *P* values between tissue types, however, may be due to the limited sample size of some tissues. This notion is consistent with the observation that three tissue types with larger sample size (blood, *n* = 92; liver, *n* = 68; and skin, *n* = 105) showed more consistent aging effects on DNA methylation (Fig. 2a,b and Extended Data Fig. 3).

To identify top genomic loci that exhibited age-related methylation changes in each tissue, a threshold of *P* < 10^−5^ was adopted. Genes that are proximal to the top-scoring age-related CpGs in each tissue are as follows: blood, *LHFPL4* (CpG is located in an exon, Fisher-z transformation of correlation (*z*) = 13.4); liver, *BARHL2* upstream (*z* = 15.3); skin, *PLD5* exon (*z* = 17.5).

**Extended Data Fig. 4 ∣:**
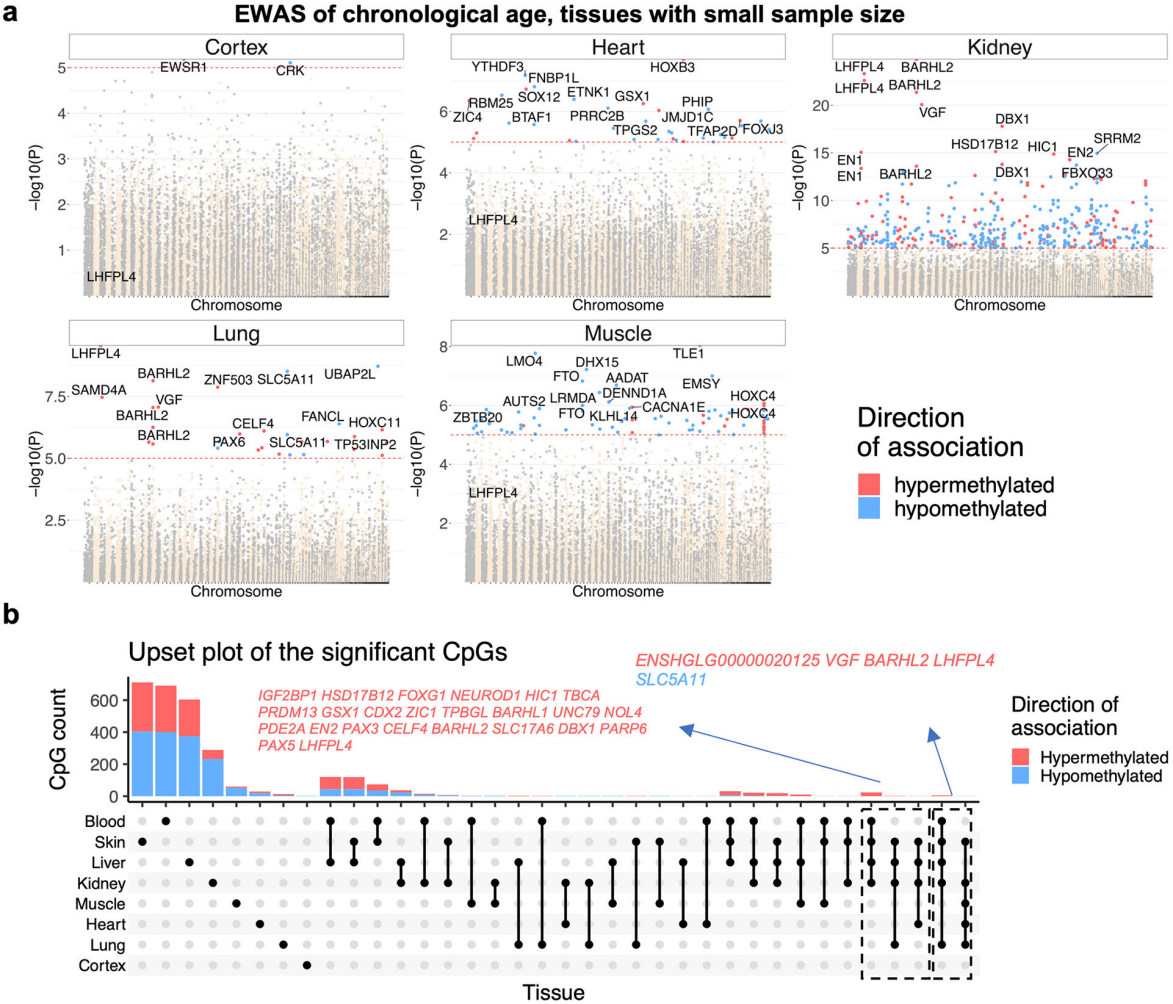
EWAS of age in NMR tissues with low sample size. **a**, Manhattan plots of the EWAS of chronological age in tissues with small sample size (cortex, 15; heart, 21; kidney, 33; lung, 19; muscle, 19). The EWAS of was done by a Pearson correlation test (and associated Student T test p value). The genome coordinates (x-axis) result from mapping the CpGs to the HetGla_female_1.0.100 genome assembly. The direction of associations with *p* < 10^−5^ (red dotted line) is color coded in red (age related increase in methylation) and blue (age related decrease). Top 15 age related CpGs are labeled by the neighboring genes. **b**, Upset plot representing the overlap of aging-associated CpGs based on individual tissues.

The EWAS results in kidney and lung tissue also implicate *LHFPL4* as one of the top hits (Extended Data Fig. 4). To identify common age-related CpGs across different tissues, an ‘upset’ plot analysis was carried out. An upset plot is a generalization of Venn diagrams to large numbers of sets. We identified CpGs with conserved age-related methylation change in at least five NMR tissue types (Supplementary Table 2). Genes that are proximal to these CpGs include *LHFPL4*, *BARHL2*, *SLC5A11* and *VGF*. We are mindful of the fact that the varying samples sizes of different tissues result in varying statistical power, which may influence the results. Hence, for a more robust identification of shared age-related CpGs, we limited this analysis to the three tissues (blood, skin and liver) for which there were more than 50 samples. This identified 60 common loci in the NMR genome that have consistent age correlations in the three tissues (Fig. 2b).

**Fig. 3 ∣:**
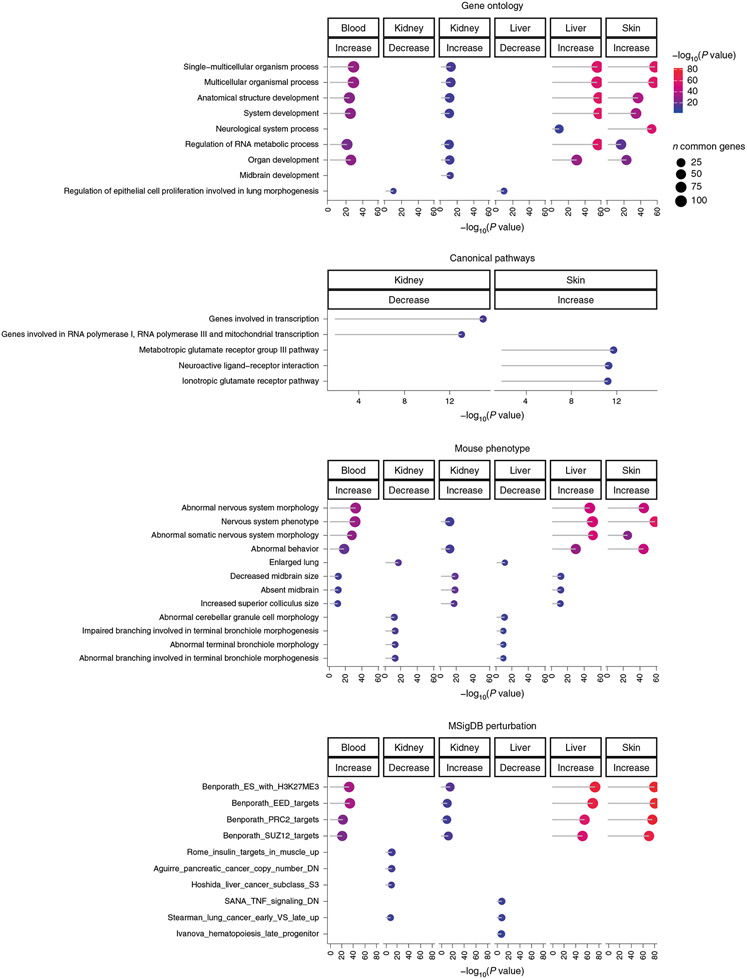
Enrichment analysis of age effects on methylation levels in NMR tissues. We used GREAT^52^ with a human Hg19 background. To protect against biases, we chose a suitable background in the enrichment test. Specifically, the background was defined as the set of 14,764 CpGs that could be mapped to the same gene in both species (NMR and human). We report the top three enriched terms/pathways for each category (canonical pathways, diseases, gene ontology, human/mouse phenotypes, promoter motifs and molecular signatures database) that were significant at a nominal (uncorrected) GREAT test *P* < 10^−10^.

To gain additional insights into age-related biological pathways, we performed enrichment analyses. Enrichment analysis of genes adjacent to age-related CpGs in tissue types with sufficiently large sample size, and also the shared 61 CpGs, revealed the potential involvement of several canonical pathways that are known to be related to the biology of aging. These include regulation of telomerase, FOXA1-related pathway, mitochondrial function and Wnt signaling. Other pathways highlighted were mostly related to regulation of transcription in general. This is consistent with the results of gene ontology (GO) analysis in which DNA binding featured prominently amongst other processes (Fig. 3 and Supplementary Table 3). Furthermore, the genes proximal to hypermethylated age-related CpGs are locations of H3K27Me3 marks, polycomb protein EED binding sites and PRC2 targets. It is acknowledged that CpG methylation can also influence expression of more distal genes. The absence of any means to identify these genes, however, precludes their analysis.

### Transcriptomic and proteomic data in liver.

Since age-related methylation changes do not overlap with age-related transcriptomic changes in human bulk tissue^16,17^, we hypothesized that the same applies to the NMR. To test this hypothesis, we intersected genes implicated by our EWAS of age in liver with age-related genes implicated by transcriptomic and proteomic studies of NMR liver samples (*n* = 6)^18^. While our EWAS implicated 1,076 genes (Wald test false discovery rate (FDR) = 10^−4^, nominal *P* < 10^−5^), transcriptional data implicated 4,287 age-related genes (FDR = 0.05) and the proteomic data implicated 484 age-related genes (FDR = 0.05).

The set of 1,076 EWAS genes did not overlap significantly with genes implicated by transcriptomic or proteomic data (hypergeometric *P* > 0.2). The transcription levels of 202 of 1,076 EWAS genes correlated significantly (FDR = 0.05) with age in NMR liver (Fig. 2e). Only seven genes (that is, *ACSS2*, *ATP5L*, *CHTOP*, *EWSR1*, *F12*, *LRPPRC*, *TOE1*) related to age according to all three data types: DNAm, transcription and protein levels.

Previous proteomic studies of the NMR and the phylogenetically closely related, shorter-lived guinea pig revealed a unique signature of mitochondrial proteins^18^. Our liver methylation studies of long-lived species (NMR, vervet monkey) versus short-lived species (mouse) corroborate this finding: significant enrichments could be observed for genes related to mitochondrial morphology (uncorrected hypergeometric *P* = 0.0070, genes *EN1*, *FOXG1*, *POLG*) and abnormality of mitochondrial metabolism (*P* = 0.0011, *ACADVL*) (Supplementary Table 4).

### Age effects in primates, mouse and NMR tissues.

As mentioned above, a major unique feature of NMRs is their outstanding longevity compared with other small rodents, such as the mouse. Humans are another species with an extraordinarily long lifespan. As such, NMRs and humans are joint outliers with regards to expected lifespan. Hence, it is of interest to identify age-related CpGs that are shared between NMRs and humans, but not with mice, which are phylogenetically closer to the NMR. The identification of genes that are proximal to these CpGs may enlighten us about the molecular and cellular processes that underpin the longevity of NMRs and humans. Towards this end, we compared age-associated CpGs in three tissues (liver, skin and blood), for which there were sufficiently large numbers of samples in all three species (Supplementary Table 1). We did not, however, utilize human liver data because the human liver samples in our possession were from older adults while many NMR livers were from young animals. This would introduce a bias that would have affected the results. As such, we used liver data from another primate: the vervet monkey, whose liver samples were profiled uniformly across the entire lifespan^24^. The vervet monkey exhibits a substantially longer maximum lifespan than the house mouse (30.8 yr versus 4 yr according to the ‘anAge’ database^25^).

**Extended Data Fig. 5 ∣:**
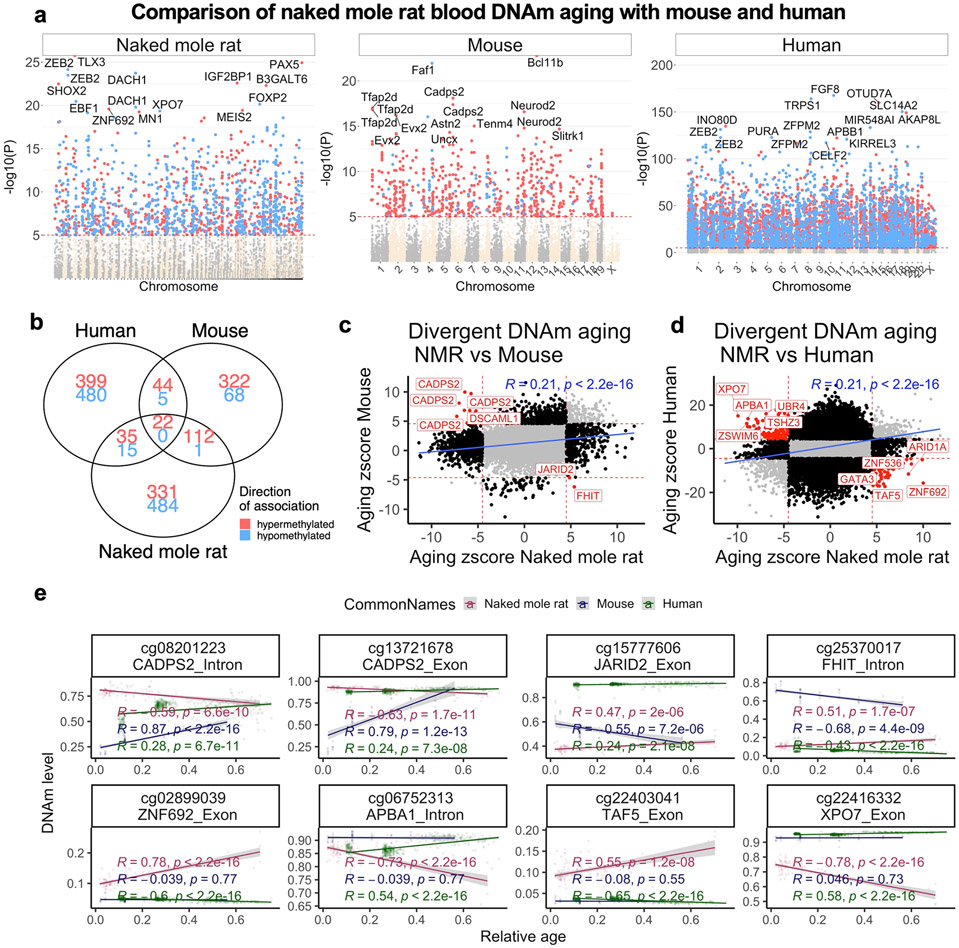
Blood methylation data from the naked mole-rat, mouse and humans. **a**, Manhattan plots of DNA methylation aging loci in NMR, human and mouse. The red line in the Manhattan plot indicates p < 10^−5^. The analysis was limited to 11,814 probes that aligned to orthologous genes in all three species. Sample sizes: NMR, n = 92; Mouse, n = 58; and human, n = 511. The red line in the Manhattan plot indicates *p* < 10^−5^. The EWAS was done by a Pearson correlation test of DNAm and Age for each species. **b**, Venn diagram of the overlap of significant CpGs (p < 10^−5^) in three species. Scatter plots of aging effects (correlation test Z statistics) in **c**, NMR (x-axis) and mouse (y-axis), and **d**, NMR and humans. The Z statistics result from applying the Fisher z-transformation to the Pearson correlation between CpG and age in the respective species. Positive/negative values of the Z statistic indicate an age related increase/decrease in methylation. **e**, The highlighted CpGs exhibit diverging in aging patterns between NMR and other species (red=NMR, blue=mouse, green=human). Each dot corresponds to a tissue sample. DNA methylation levels (y-axis) versus relative age (x-axis) for select CpGs with divergent aging patterns between the species. R, Pearson correlation coefficient. P, correlation test p-value.

**Extended Data Fig. 6 ∣:**
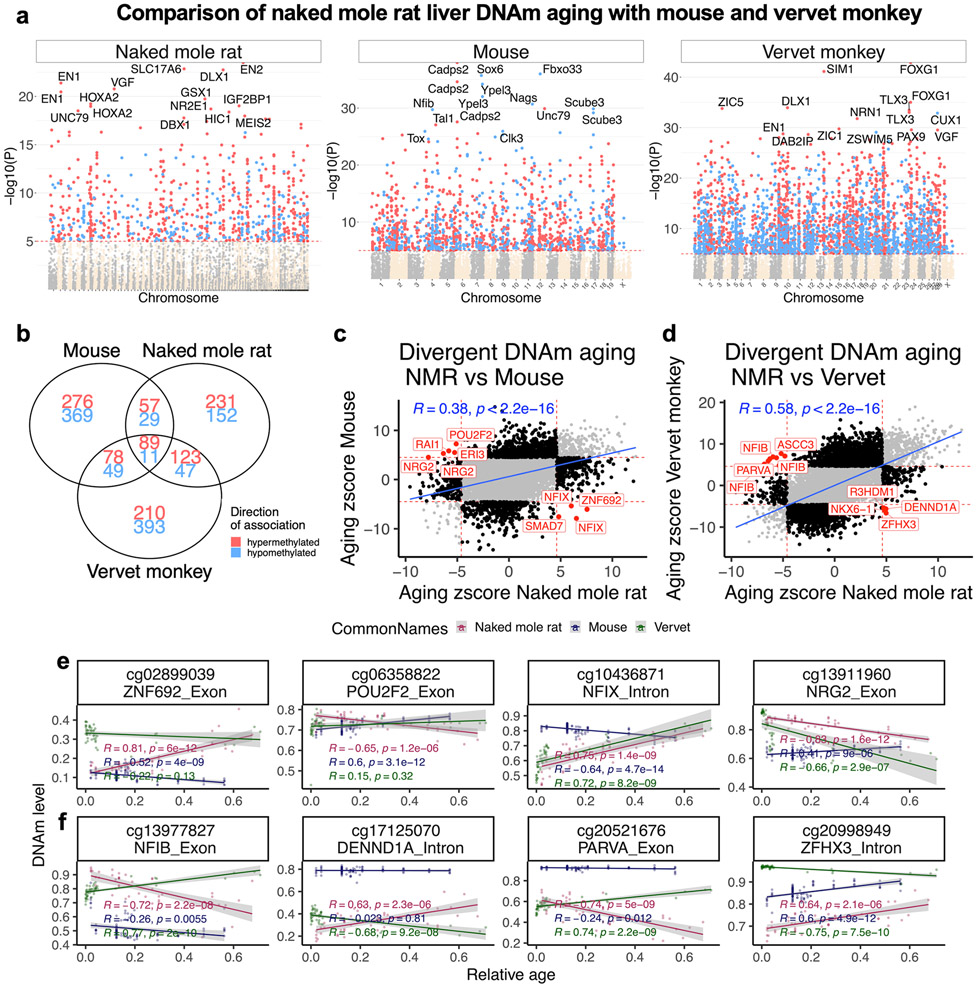
Liver from NMR, mouse and vervet monkey. **a**, Manhattan plots of DNA methylation aging loci in NMR, vervet monkey and mouse. The red line in the Manhattan plot indicates p<10^−5^. The analysis was limited to 11,793 probes that aligned to orthologous genes in all three species. Sample sizes: NMR n = 62; Mouse n = 111; and vervet monkey n = 48. The red line in the Manhattan plot indicates p<1e-5. The EWAS was done by a Pearson correlation test of DNAm and Age for each species. **b**, Venn diagram of the overlap of the up to 1000 top CpGs (500 in each direction) that are significantly associated with age (p<10^−5^) in the three species. Scatter plots DNA methylation aging effects in **c**, NMR (x-axis) and mouse and **d**, NMR and humans. The Z statistics result from applying the Fisher z-transformation to the Pearson correlation between CpG and age in the respective species. Positive/negative values of the Z statistic indicate an age related increase/decrease in methylation. The highlighted CpGs exhibit diverging in aging patterns between NMR and other species (red=NMR, blue=mouse, green=vervet monkey). Each dot corresponds to a tissue sample. DNA methylation levels (y-axis) versus relative age (x-axis) for select CpGs with divergent aging patterns between **e**, NMR and mouse and **f**, NMR and vervet. R, Pearson correlation coefficient. P, correlation test p-value.

**Fig. 4 ∣:**
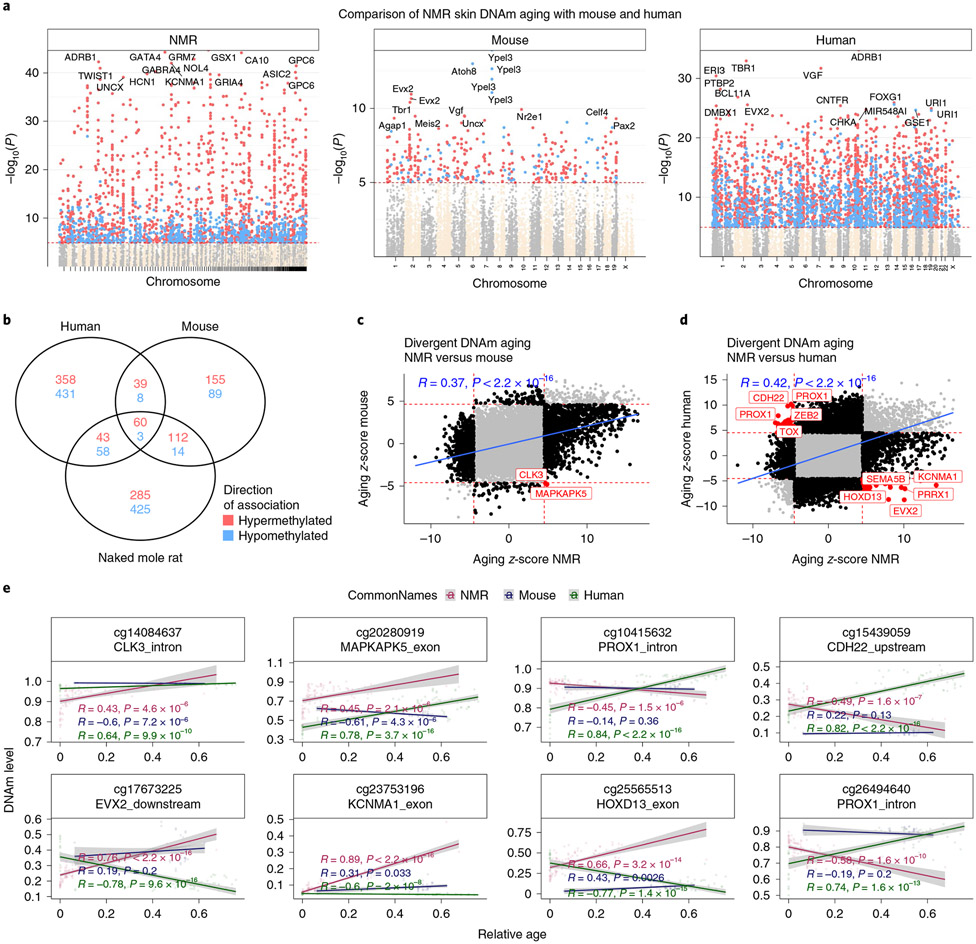
Skin methylation data from the NMR, mouse and human. **a**, Manhattan plots of DNA methylation aging loci in NMR, human and mouse. The red line in the Manhattan plot indicates *P* < 10^−5^. The analysis was limited to 11,814 probes that aligned to orthologous genes in all three species. Sample sizes: NMR, 104; mouse, 42; and human, 72. The red line in the Manhattan plot indicates *P* < 10^−5^. The EWAS was done by a Pearson correlation test of DNAm and age for each species. **b**, Venn diagram of the overlap of significant CpGs (*P* < 10^−4^) in three species. **c**,**d**, Aging effects on cytosine methylation in the NMR (*x* axis) versus those in mouse (*n* = 42 mouse skin samples) (**c**) and human (**d**). Each dot corresponds to a cytosine. Each axis reports the *Z* statistics resulting from applying the Fisher *z*-transformation to the Pearson correlation coefficient between cytosine methylation and age in the respective species. A positive or negative value of the *Z* statistics corresponds to an age-related increase or decrease, respectively. The highlighted CpGs (red color) correspond to cytosines with distinct aging patterns in the NMR compared with the other species. **e**, Select CpGs with divergent aging patterns in NMR compared with the other two species. The red dots and red regression line correspond to NMR samples. Blue and green dots correspond to mouse and human skin samples, respectively. *R*, Pearson correlation coefficient; *P*, correlation test *P* value.

In general, we observed that age-related CpGs of NMRs overlapped moderately with those of mice and primates (human and vervet monkey); age-related CpGs of NMR blood correlated weakly with those of mouse (Pearson correlation *r* = 0.21) and human (*r* = 0.21, Extended Data Fig. 5). Stronger correlations between age-related CpGs of NMRs could be observed for skin (*r* = 0.39 for mouse and *r* = 0.43 for human, Fig. 4) and liver (*r* = 0.38 for mouse and *r* = 0.58 for vervet monkey, Extended Data Fig. 6).

**Extended Data Fig. 7 ∣:**
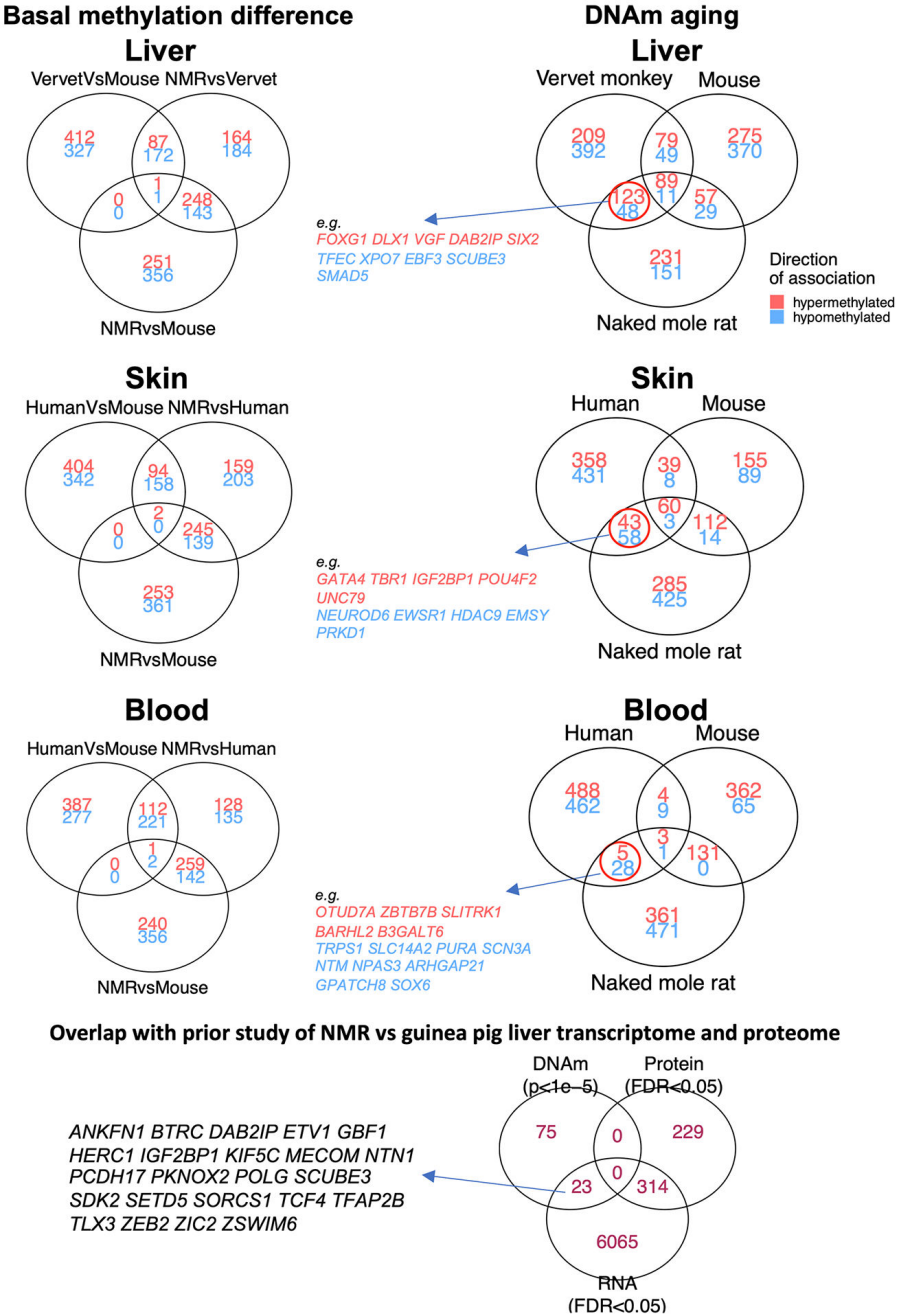
DNAm aging signatures that are shared between NMR and primates but not with mouse. Venn diagrams represent the overlap of top CpGs in different EWAS analysis. The left column shows a pairwise basal (mean) DNAm difference between NMR, primates and mouse. The right column shows the overlap of top CpGs with DNAm aging between NMR, primates and mouse. We identified the changes that were shared between NMR and primates, but not in mouse. These CpGs are reported in Supplementary Table 12.

**Fig. 5 ∣:**
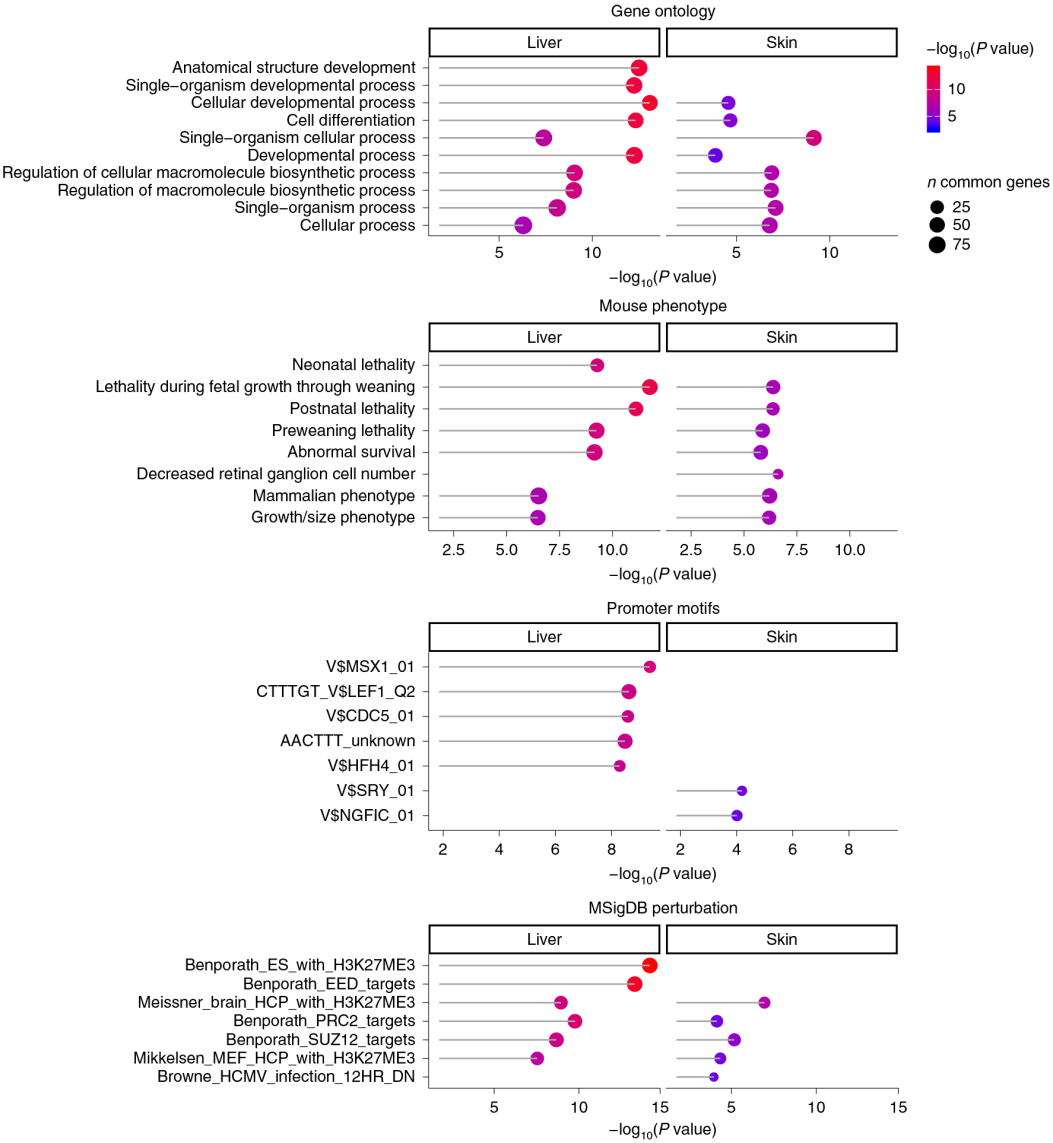
Enrichment analysis of the tissue-specific DNAm signatures that are shared between NMR and primates, but not with mouse. The input includes the CpGs that had a consistent aging pattern between the two long-lived species (NMR and primate) but a divergent aging pattern in the short-lived species (mouse). Enrichment *P* values were estimated using GREAT^52^. The gene level enrichment was done using GREAT analysis^52^ and human Hg19 background. As background set, we chose 11,814 human Hg19 background locations/probes that mapped to the same gene in the NMR, human and mouse genomes. The top five enriched terms/pathways for each category (canonical pathways, diseases, gene ontology, human and mouse phenotypes, and promoter motifs) were presented if their enrichment *P* value was significant at <1 × 10^−4^.

To identify age-related CpGs associated with species longevity, we focused on those that change with age similarly between NMRs and primates, but differentially in the mouse. In total, subsets of 171 such CpGs were detected in liver, 33 in blood and 116 in skin (Extended Data Fig. 7). Identification of genes that are proximal to these cytosines revealed several that are of particular interest. Some of these that were identified in at least two of the three tissues include: hypermethylation of the *IGF2BP1* promoter, *PAX2* upstream, *SLITRK1* promoter and *B3GALT6* exon. Strikingly, these regions were highly enriched in pathways related to lethality and abnormal survival in the mouse phenotype database (Fig. 5). In general, a prominent feature of age-related CpGs that are shared between NMRs and primates, but not the mouse, is their proximity to genes that encode proteins with homeobox domains, particularly the NKL and HOXL families of proteins (Supplementary Table 12). These proteins are primarily transcription factors that regulate expression of developmental genes. As such, it follows that their perturbations in mice were reportedly associated with lethality during fetal growth, postnatal lethality, abnormal survival, decreased body size, premature aging and death, nervous system abnormality and so on (Supplementary Table 13).

Besides development, genes encoding metabolic proteins were also found to be proximal to age-associated CpGs shared between NMRs and primates but not the mouse. Collectively, these results suggest that differences in development and metabolism may, in part, jointly underlie the longevity of NMR and primates (especially humans), which is longer than expected from their body size.

We caution the readers that CpGs that are related to species longevity may not play a causal role in the aging process. However, 25 of the implicated genes (Supplementary Table 14) play a role in aging according to the Aging Gene Database (GenAge)^26^. In particular, three genes (*POLG*, *TFAP2A*, *KCNA3*) are known to play a role in human aging conditions.

CpGs that change with age in NMR and vervet monkey liver samples but not in mouse liver are presented in Supplementary Table 15. Our liver methylation studies of long-lived species (NMR, vervet monkey) versus short-lived species (mouse) finds significant enrichments for genes related to mitochondrial morphology (uncorrected hypergeometric *P* = 0.0070, genes *EN1*, *FOXG1*, *POLG*) and abnormality of mitochondrial metabolism (*P* = 0.0011, *ACADVL*) (Supplementary Table 13).

### Queens age more slowly.

NMRs, together with Damaraland mole-rats, have been proposed to be eusocial mammals. Eusocial species have very organized lifestyles and clear reproductive division of labor and roles, with the queens (the dominant breeding female in the colony) being the most distinct members of the social group. Given the genetic similarity between queens and nonbreeders, it is highly likely that eusocial characteristics could be determined by, or correlated with, epigenetic differences. Hence, we investigated the potential relationship between queen status and DNA methylation. As we had blood samples from 18 queens, we carried out this analysis primarily with blood DNA, and a subset of analyses also included skin, for which there were only four queen samples.

**Fig. 6 ∣:**
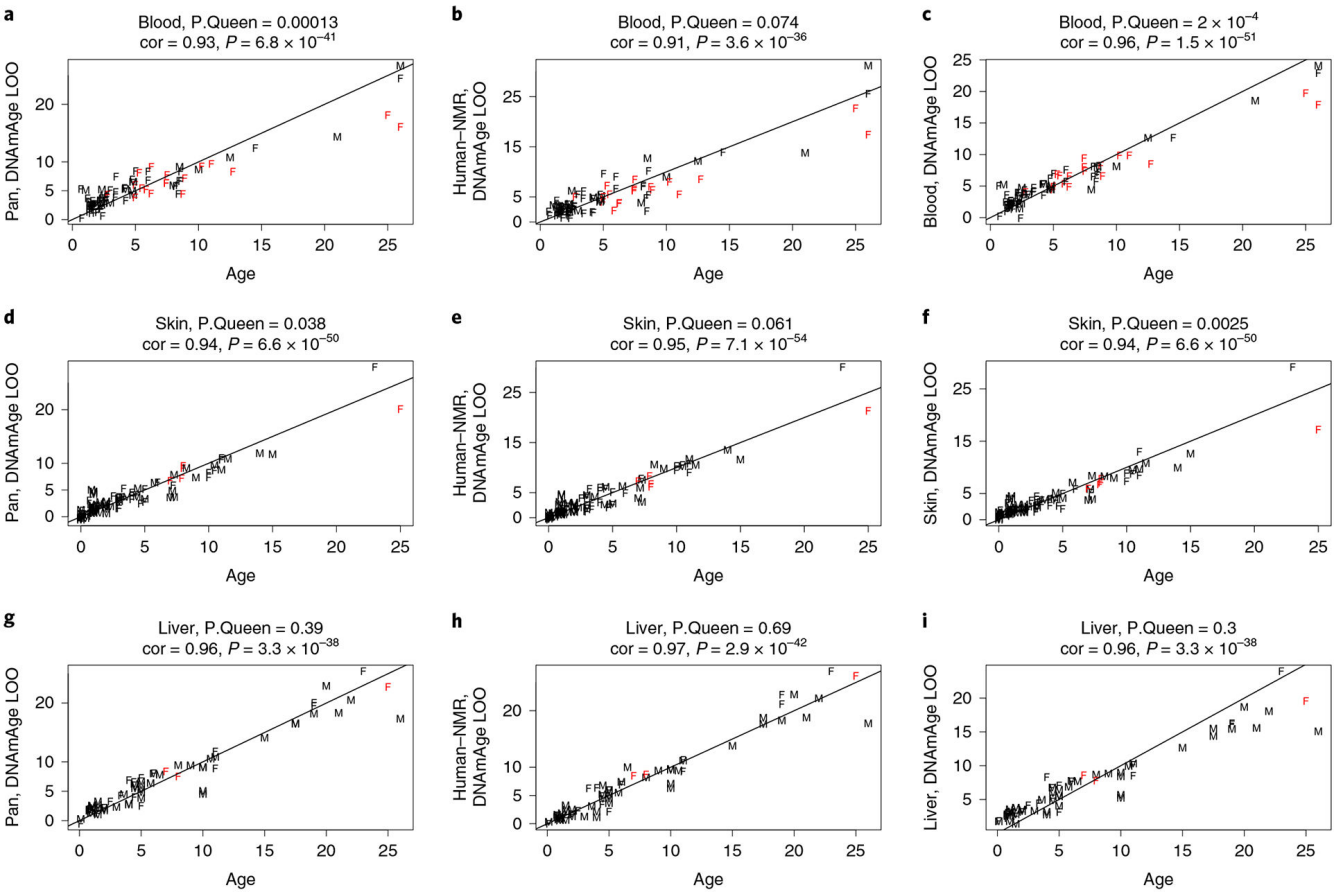
Leave-one-out estimates of DNAm age (*y* axis) colored by queen status. The chronological age (*x* axis, in units of years) at the time of the sample collection. Dots are labeled by sex (female or male) and colored by queen status: red, queen; black, nonbreeding female, male or unknown. Each row corresponds to a tissue: **a**,**b**,**c**, blood (total *n* = 92 including 18 queens); **d**,**e**,**f**, skin (total *n* = 107 including 5 queens); **g**,**h**,**i**, liver (*n* = 70 including 3 queens). Columns correspond to different epigenetic clocks: first column, **a**,**d**,**g**, results for the NMR pan-tissue clock. **b**,**e**,**h**, Results for the human–NMR clock of chronological age. **a**, Pan tissue clock applied to blood. **b**, Human-NMR clock for age applied to blood. **c**, Blood clock applied to blood. **d**, Pan tissue clock applied to skin. **e**, Human-NMR clock applied to skin. **f**, Skin clock applied to skin. **g**, Pan tissue clock applied to liver. **h**, Human-NMR clock applied to liver. **i**, Liver clock applied to liver. (**c**) (**f**) (**i**). *P.Queen* denotes the *P* value for a differential slope analysis comparing aging patterns in queens (red dots) with the remaining samples (black dots). This *P* value was calculated from a linear regression model whose dependent variable (DNAm based age estimate) was regressed on age, queen status and their interaction effect. P.Queen was calculated using the Wald test statistic for the interaction effect. F, female; M, male.

With the highly accurate epigenetic clocks in hand, we were well-placed to ascertain whether queens (that is, reproducing females) differ from female and male nonbreeders with regards to epigenetic aging rates. When tested, queens were indeed found to age more slowly than nonbreeders, as measured by leave-one-out estimates of DNAm age for blood and skin (Fig. 6). This was particularly significant in blood samples, for which we had more queen samples than for other tissues (*P* = 0.00013 with pan-tissue clock Fig. 6a; *P* = 0.074 with human–NMR clock, Fig. 6b; *P* = 0.0002 with blood clock, Fig. 6c). There was nominally significant evidence of slower aging rates in skin samples from four queens, the oldest of which showed substantially younger epigenetic age as predicted by the skin clock (Fig. 6) (*P* = 0.038 for the pan-tissue clock, Fig. 6d; *P* = 0.061 for the human–NMR clock, Fig. 3e; *P* = 0.0025 for the skin clock). The most pronounced difference between queens and nonreproducing females could be observed for older animals (Fig. 6a-f), which may reflect that older queens have been queens for a long time. However, we cannot formally test this hypothesis due to the low number of older queens and due to a lack of information surrounding the exact length of time in queen status.

**Extended Data Fig. 8 ∣:**
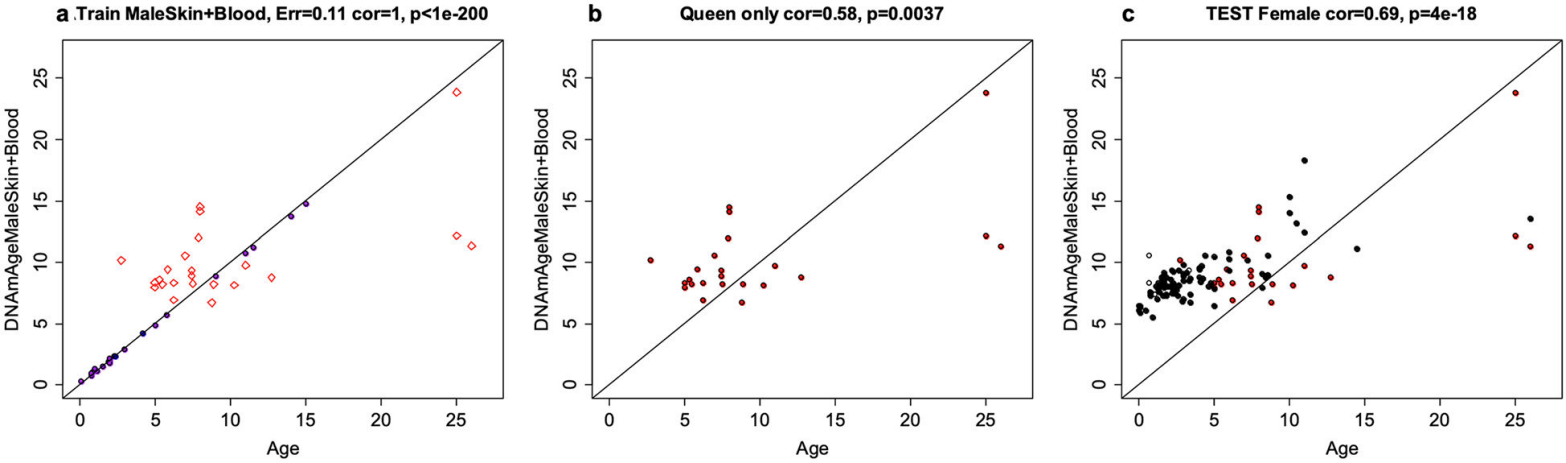
Epigenetic clock analysis of queen status in female skin and blood samples. **a-c**, Epigenetic clock trained on male skin and blood samples. **a**, Training set. The black samples correspond to the training data, that is blood and skin samples from male NMRs. Red diamonds correspond to part of the test data: blood and skin samples from queens. The latter data are also shown in a separate plot: panel **b**. **c**, Entire test data set. female skin and blood samples from queens and female laborers. Dots are colored by queen status: red=queen, black=non reproducing females, white=queen status is unknown. A multivariate linear regression model in female samples reveals that queen status is associated with lower epigenetic aging (p=0.0148) according to the male NMR clock.

Age estimates of other tissues such as liver were not significant and inconclusive due to even smaller sample size (Fig. 6g-i). The above-mentioned analysis has a statistical limitation: both queens and nonqueens were used to arrive at the leave-one-out estimates of DNAm age. Since the training sets contained queens, the resulting clock may inadvertently condition out the slower aging effects of queens. To address this concern, we employed the second analysis strategy. We used male samples as training data and female data as test data. Thus, we first developed an epigenetic clock for blood and skin from male NMRs (training set). We then used this male clock to evaluate female blood and skin samples (female test set). Queens were once again observed to exhibit slower epigenetic aging, according to visual inspection (Extended Data Fig. 8) and by multivariate linear regression model analyses of female samples (Wald test *P* = 0.015). Since we were concerned that samples from older queens may drive the observed association, we repeated the analysis in animals who were younger than 15 yr. Again, a multivariate regression analysis revealed that queens aged more slowly than female nonqueens (*P* = 0.031).

Male breeding status was not available for skin and blood tissues. In liver samples, we did not observe a significant association (Wald test *P* > 0.2) between male breeding status and epigenetic aging rates, which may reflect the low sample size (only *n* = 4 known male breeders versus *n* = 27 nonbreeding males).

**Fig. 7 ∣:**
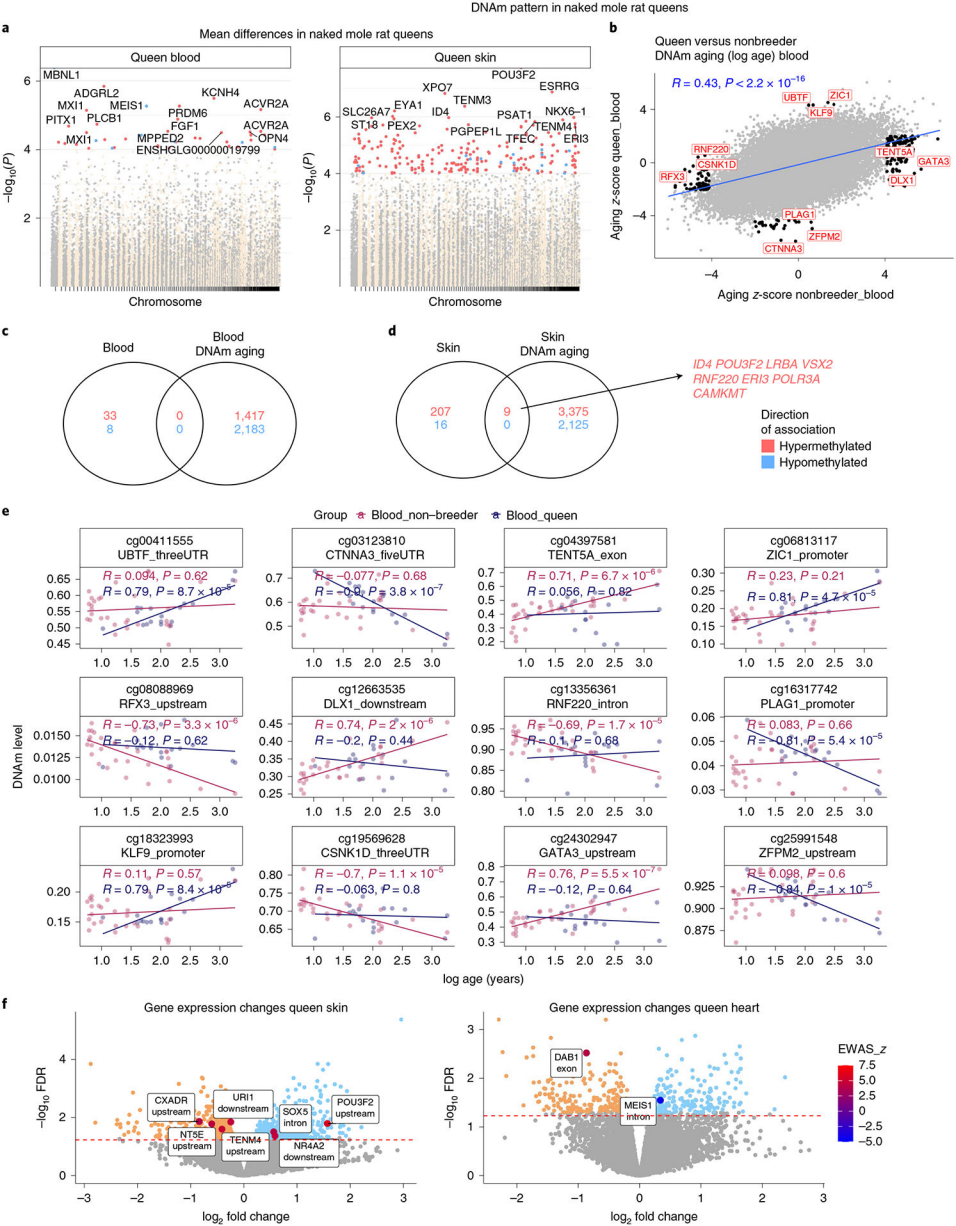
Queen status modestly alters DNA methylation pattern in NMR. **a**, Manhattan plot of the EWAS of queen status (queen versus nonbreeder females) in blood and skin of NMR. A multivariate regression model was used to regress each cytosine (dependent variable) on queen status and chronological age. The *y* axis reports log_10_-transformed Wald test *P* values associated with queen status. Sample sizes: nonbreeding female blood, *n* = 32; nonbreeding female skin, *n* = 8; queen blood, *n* = 18; queen skin, 4. The red line in the Manhattan plot corresponds to a significance level of *P* = 1 × 10^−4^. **b**, Sector plot for finding CpGs (dots) with distinct epigenetic aging patterns in the blood of queens compared with that of nonbreeding females. We used log-transformed (base e) age since the age distribution of queens was highly skewed. Each axis reports the *Z* statistics resulting from an EWAS of log-transformed (base e) age in queens (*y* axis) and nonbreeding females (*x* axis). The *Z* statistics are the Fisher *z*-transformations of the Pearson correlation. A positive value of the *Z* statistics indicates that cytosine methylation increases with age. The black dots indicate CpGs that change in one group (*P* < 10^−4^, red dotted line) but not in the other (*P* > 0.05, blue dotted line). Skin tissue was excluded from this analysis due to limited sample size for a stratified EWAS of age. **c**,**d**, Venn diagrams of the overlap between queen effects in blood (**c**), skin (**d**) and CpGs with a significant correlation with age in each tissue. **e**, Aging patterns in blood samples for select CpGs that differ between queens and nonbreeding females. Methylation levels (beta value, *y* axis) versus chronological age. Blue dots and the blue regression line visualize results for queens. Red dots visualize results for nonbreeding females. *R*, Pearson correlation coefficient; *P*, correlation test *P* value based on the Student’s *t*-test. **f**, Volcano plot of publicly available gene expression data from NMR skin and heart (Gene Expression Omnibus GSE98663 (ref. ^27^)). log_2_-transformed fold change (*x* axis) versus log_10_-transformed FDR in skin and heart tissue samples. Positive values on the *x* axis correspond to genes that are over-expressed in queens compared with female nonbreeders. The gene symbols indicate genes that were also implicated by our methylation study.

We identified 41 CpGs in blood and 232 in skin that were differentially methylated between queen and nonbreeding females at a significance of α = 10^−4^ (Fig. 7a and Supplementary Tables 16 and 17). Many of the genes proximal to identified CpGs are in regions that encode transcription factors that are involved in neural/brain processes, which may possibly contribute to differential behavior between queens and nonbreeders. These sets of genes will be of interest for researchers investigating NMR behavior differences between queen and nonbreeders.

Age-related CpG methylation changes in queen blood were found to correlate well (*R* = 0.43) with those of nonbreeder females (Fig. 7b), indicating broad conservation of epigenetic aging mechanisms between them, despite the differences in aging rate. However, we did identify a total of 237 age-related CpGs with unique methylation patterns in either of these groups (Fig. 7b,e and Supplementary Table 18). It is possible that such mutually exclusive age-related CpGs may contribute to the different rate of epigenetic aging between queens and nonbreeders. This remains to be empirically tested, although enrichment analysis of these 237 unique age-related CpGs highlighted the involvement of developmental pathways, particularly those of sensory perception (Supplementary Table 19).

**Fig. 8 ∣:**
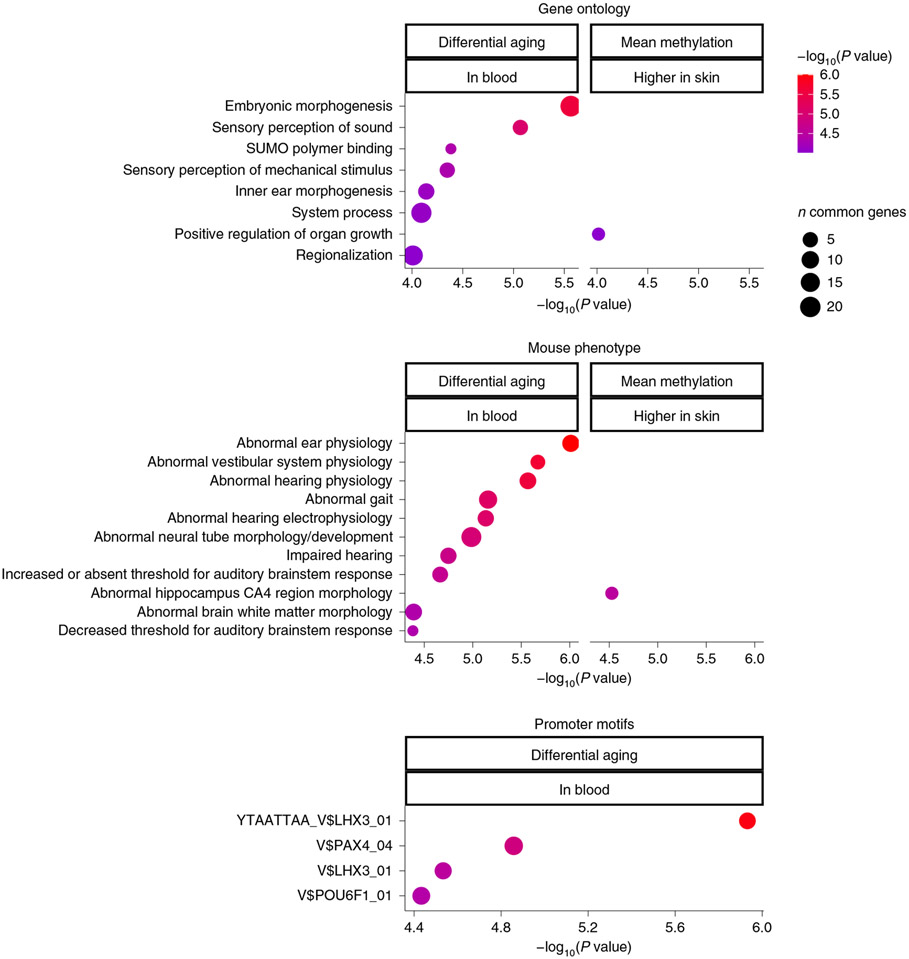
Gene set enrichment analysis of NMR female queen status basal effects and CpGs with distinct DNA methylation aging. The enrichment *P* values were calculated with GREAT^52^. The columns correspond to different inputs: differential aging/blood denotes the set of CpGs whose aging pattern in blood differs between queens and nonbreeding females. Mean methylation/higher in skin denotes CpGs whose mean methylation in queens is significantly higher than in nonbreeding females after correcting for age. The GREAT analysis^52^ used 14,764 background locations/CpGs that mapped to the same gene in both species (NMR and human Hg19). The top 10 enriched datasets from each category (gene ontology, human and mouse phenotypes, and upstream regulators) were selected if they were significant at a nominal *P* < 10^−4^.

A transcription factor enrichment analysis of differential aging rates in blood identified the LHX3 motif (*P* = 10^−6^, Fig. 8). LHX3 transcription factor controls pituitary development and may be responsible for maintaining both reproductive status and aging characteristics of the animals.

In skin, we identified nine CpGs that were associated with queen status as well as age (Fig. 7d). These CpGs could potentially contribute to epigenetic age differences in the skin of these two groups of NMRs, although it is necessary to be mindful of the limited number of skin samples and the fact that we did not identify in blood any age-related CpGs with baseline methylation that differed between queens and nonbreeders (Fig. 7c). Collectively, the various analyses above have engendered an interesting list of target genes to be tested for their potential contribution in regulating NMR aging, queen status and behavior.

**Extended Data Fig. 9 ∣:**
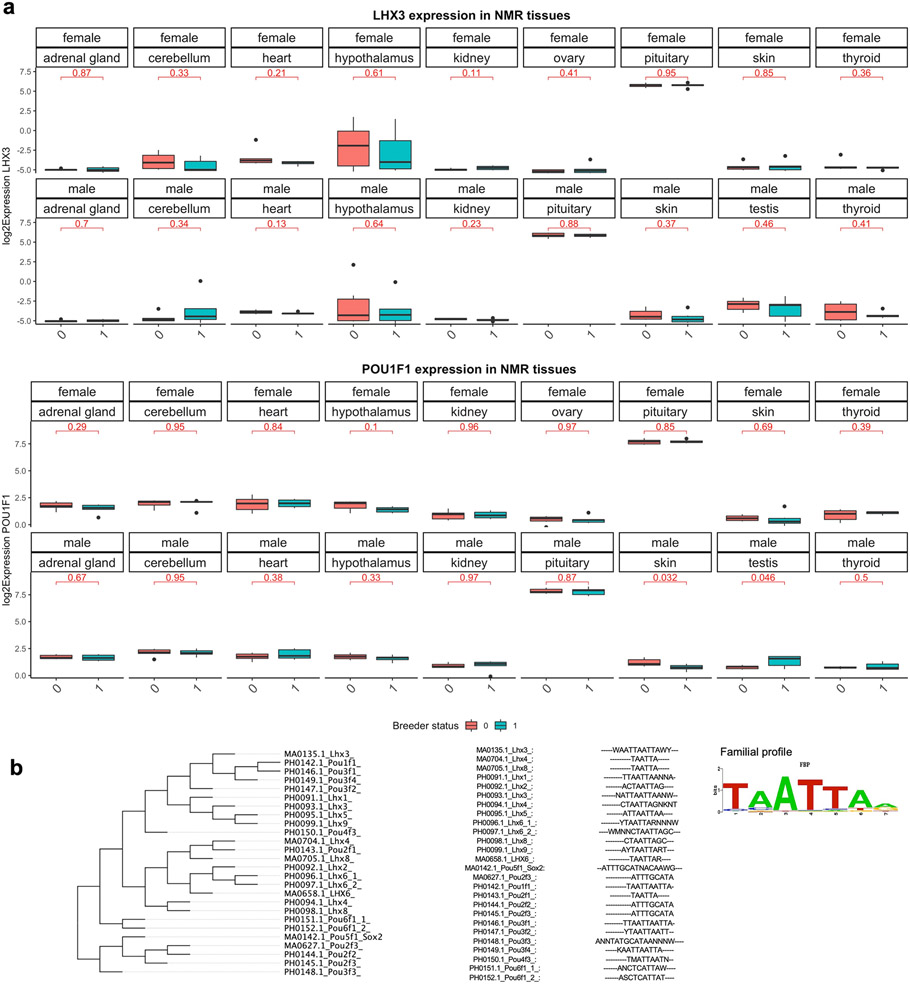
Gene expression of LHX3 and POU1F1 versus female and male breeding status in NMR tissues. **a**, LHX3 and POU1F1 are two transcriptional factors that interact with the LHX3 motif. Using publicly available data (Gene Expression Omnibus GSE98663) from Bens et al 2018, we found no significant association between LHX3, POU1F1 and female queen status but male breeding status was associated with lower POU1F1 expression levels in skin (uncorrected two-sided Student T test p=0.032) and higher expression levels in testis (p=0.046). The box plots show the median, lower quartile (Q1), upper quartile (Q3) of the data for each group. The whiskers show maximum and minimum calculated by 1.5*Interquartile range+Quartiles. The notches indicate 95% confidence intervals of the median. N=6 breeder and non-breeders per tissue. The mean differences in plots are calculated by two-sided Student T-test. **b**, Multiple sequence alignment of LHX and POU families of transcriptional factor motifs in Mus musculus. Lhx3 motif has high similarity with Lhx1, Lhx9, Lhx5, Pou1f1, Pou3f4, and Pou3f2 motifs. The alignment was done using STAMP tool and default settings which are as follows. Column comparison matrix: Pearson correlation; Alignment method: Smith-Waterman (ungapped & unextended); tree building method: unweighted pair group with arithmetic means; multiple sequence alignment: progressive profile alignment.

We related our findings to those from a previous transcriptomic analysis of queen status in the NMR^27^. The overlap between queen-related genes implicated by our EWAS and those implicated by the transcriptomic analysis was insignificant (hypergeometric *P* > 0.20). However, nine genes relate to queen status according to both data types in skin (*POU3F2*, *SOX5*, *TENM4*, *CXADR*, *USI1*, *NR4A2*, *NT5E)* and heart tissue (*DAB1*, *MEIS1*) (Fig. 7f). Male breeders have a longer lifespan compared with nonbreeders as well as female breeders (queens)^11^. The pituitary gland and LHX3 are important for male sexual maturation as well but we could not carry out an EWAS of age in male breeders. However, we carried out a transcriptional study of male and female breeding status using publicly available data (Gene Expression Omnibus GSE98663) from refs. ^27,28^. POU1F1, a pituitary-specific POU-homeo-domain transcription factor, and its cofactor LHX3 were not associated with female reproduction status (Extended Data Fig. 9a), but male breeding status was associated with lower POU1F1 expression levels in skin (uncorrected two-sided Student’s *t*-test *P* = 0.032) and higher expression levels in testis (*P* = 0.046). Multiple sequence alignment of Lhx family motifs suggested high similarity of Lhx3 with Lhx1, Lhx9, Lhx5, Pou1f1, Pou3f4 and Pou3f2 motifs (Extended Data Fig. 9b). Thus, there could be other transcriptional factors that interact with this motif, particularly in tissues other than pituitary glands. For example, *POU3F2* was a gene with both DNA methylation and transcriptional changes in skin of NMR queens (Fig. 4f). Another transcriptional study of NMR brains suggested a significant increase in *Lhx9* (log_2_-transformed fold change = 3) and *Pou4f1* (log_2_-transformed fold change = 5) in queens compared with nonbreeder females^29^. Thus, all of these studies suggest that LIM (Lhx) and Pou homeobox transcription factors play a significant role in breeding behavior of female, and even male, NMRs.

**Extended Data Fig. 10 ∣:**
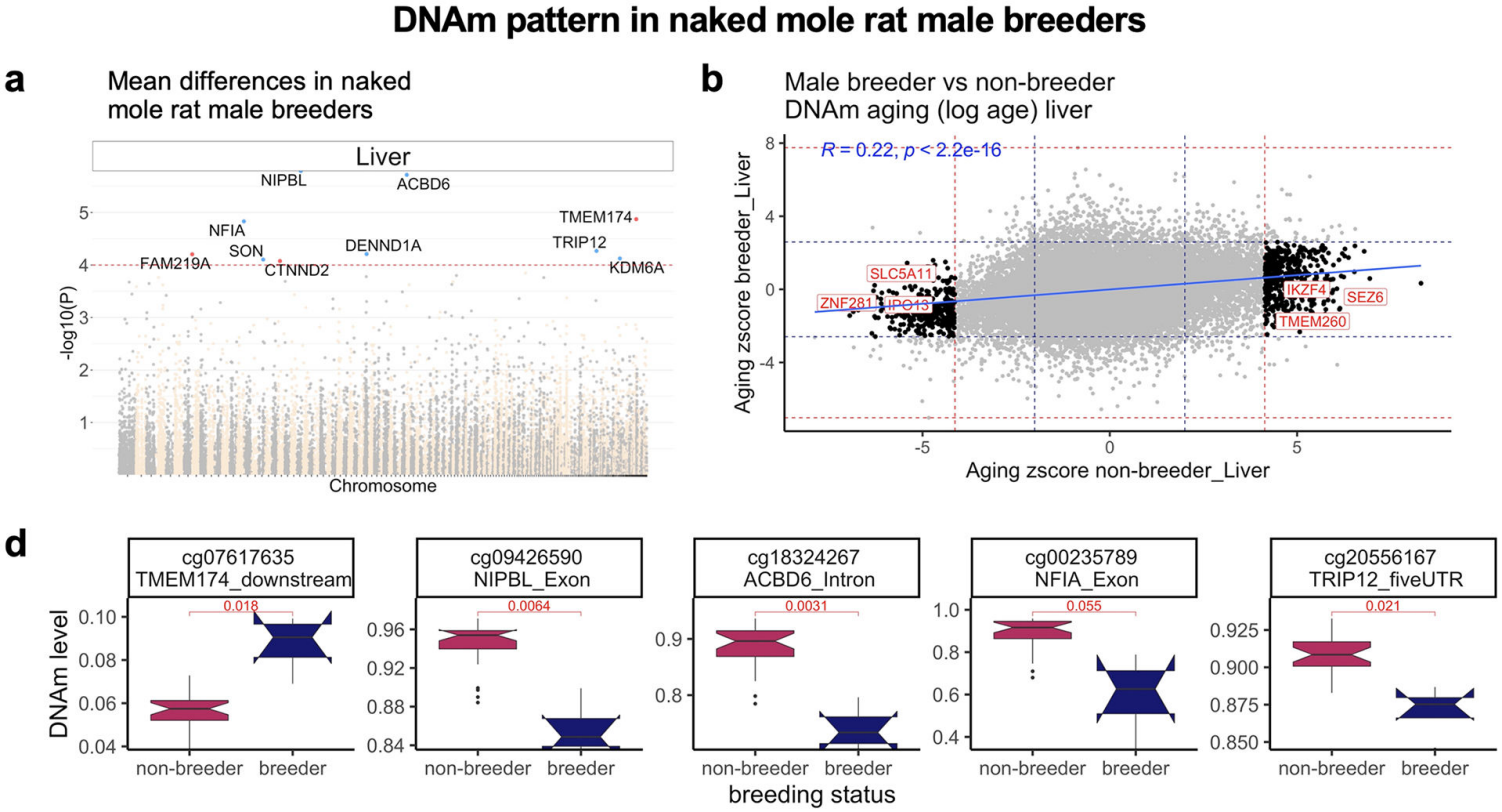
Breeding status modestly alters DNA methylation pattern in NMR males. **a**, Manhattan plot of the EWAS of breeding status (breeder vs non-breeder males) in liver of NMR. Sample sizes: N=4 breeding males versus N=27 non-breeding males. The EWAS analysis used chronological age as covariate in order to adjust for this potential confounder. The red line in the Manhattan plot corresponds to a significance level of p=1 × 10^−4^. **b**, Sector plot of the CpGs with distinct DNA methylation aging in the blood of breeder and non-breeder males. We carried out a log transformation (natural log) of age since the age distribution of queens was highly skewed. The black dots indicate the CpGs that change in one (p<10^−4^, red dotted line) and do not change (p>0.05, blue dotted line) in the other analysis. **c**, Aging effects on methylation levels in liver samples for select CpGs that differ between breeding and non-breeding males. The box plots show the median, lower quartile (Q1), upper quartile (Q3) of the data for each group. The whiskers show maximum and minimum calculated by 1.5*Interquartile range+Quartiles. The notches indicate 95% confidence intervals of the median. Two sided Student T-test p-values were used to test for differences in mean methylation levels.

Our EWAS of male breeding status in liver samples was underpowered (only *n* = 4 known male breeders versus *n* = 27 nonbreeding males). But we found suggestive evidence of hypomethylation in an exon of *NIPBL* (uncorrected Wald test *P* = 0.0064) and an intron of *ACBD6* (*P* = 0.0031, Extended Data Fig. 10). CpGs associated with female breeding status in blood and skin did not overlap significantly with those related to male breeding status in liver (hypergeometric *P* > 0.2).

## Discussion

This study describes seven epigenetic clocks for NMRs, of which five are specific to NMRs (for different tissue types) and two are dual-species human–NMR clocks that are applicable to humans as well. The human–NMR clocks for chronological and relative age demonstrate the feasibility of building epigenetic clocks for different species based on a single mathematical formula. This further consolidates emerging evidence that epigenetic aging mechanisms are conserved, at least between members of the mammalian class.

The first step towards developing these clocks was the removal of the species barrier that precluded the use of Human Illumina Methylation arrays to profile nonhuman species. In response, we used the mammalian methylation array that profiles CpGs with flanking DNA sequences that are highly conserved across numerous mammalian species. This allowed us to generate DNA methylation profiles from different species using the same DNA methylation measurement platform. This is a technical step change from previous NMR studies that utilized sequence-based measurement platforms^15,22^, which do not lend themselves to direct comparison with other species.

On a phenotypic level, the NMRs appear to evade aging. Hence, we did not know whether they display epigenetic changes with increasing age. Our study clearly detected significant age-related changes in DNA methylation levels across the entire lifespan of the animal, even in relatively young animals. This contradiction between phenotypic and epigenetic aging could imply that age-related DNA methylation changes do not matter since they do not appear to correlate with any adverse functional consequences in NMRs. However, accelerated epigenetic aging has been correlated to a very wide range of pathologies and health conditions^30^. Alternatively, it could mean that while the NMR ages at a molecular level, as do all other mammals, it has developed compensatory mechanisms that counteract the consequences of these epigenetic changes. The NMR age-related CpGs that we identified, and the availability of epigenetic clocks, are valuable resources to resolve this question.

We find that iPS cells from NMRs are very young, which mirrors what has been observed in humans^16^. In particular, our skin clock for NMRs indicates that iPS cells have a fetal age. This consolidates the notion that the clocks we developed tap into the biology of NMRs that relates to the process of aging.

Our analysis of iPSCs was limited to a single method of generating iPSCs. We used NMR iPSCs generated with four Yamanaka factors plus Large T antigen. Large T is a strong oncogene, and the iPS cells generated by using it may be different from ordinary iPSCs, which may affect their methylome. Another group reported the generation of iPSCs without Large T antigen, but the resulting iPSCs exhibited aneuploidy^31^. As reported elsewhere, we failed to generate iPSCs without the use of Large T antigen, which led us to conclude that NMRs have a more stable epigenome^23^. In a previous study, we did not observe consistent rejuvenation resulting from SV40 Large T in human fibroblasts^32^. In the case of our NMR iPSCs, however, all of the cultures tested showed strong rejuvenation consistent with the rejuvenation observed during iPSCs generation in other species, such as human and mouse^16,33^.

Further clues to NMR aging were also revealed from the three-way comparison of age-related CpGs between NMRs, primates and mice. Although primates and NMRs are phylogenetically more distantly related than NMRs and the mouse, these relationships are not similarly manifested when it comes to longevity. Indeed, NMRs and humans are more akin to each other as they are both outliers with regards to lifespan expected from their adult size. Here, the three-way comparison revealed that the reason for the unusually long lifespans of NMRs and primates may lie in the coregulation of developmental and metabolic processes. Conversely, similarly regulated developmental genes between NMR and human may reflect neotenic features characteristic of these two species^34^. Neoteny is defined as retention of juvenile features into adulthood. A shift towards longer development and retention of youthful tissue repair can lead to longevity.

With regards to NMR lifestyle, we identified a large number of CpGs in blood and skin that are differently methylated between queens and nonbreeding females. Remarkably, demographic data from a large number of captive-bred NMRs showed longer survival of breeders^11^. This is consistent with the observed slower epigenetic aging of queens, which argues for biological relevance of age-related epigenetic changes in NMRs, as was considered above. Further analysis of DNA methylation aging in the function of social status (queens versus nonbreeders) implicated LIM and POU homeobox transcription factors as potentially important aging rate determinants, which are known to play roles in both pituitary and central nervous system development^35^. LHX3 binds to promoters of several genes involved in pituitary development and function^36^, which could potentially regulate lifespan. Strikingly, alterations in pituitary development were previously shown to lead to a slow aging phenotype in Ames and Snell dwarf mice^37,38^.

Our study was limited in that we did not have access to transcriptomic data and methylation data from the same animals. Based on human studies, we expect that the *cis* relationship between methylation and gene expression will be weak in bulk tissue due to large cell-to-cell variability^39^. The study of methylation and expression changes will greatly benefit from single-cell data^40^.

In summary, we have developed robust epigenetic clocks for NMRs. These clocks can be used to estimate the age of wild NMRs, and more excitingly to facilitate the studies of NMRs as a model organism for aging, longevity and suppression of pathologies. The dual human–NMR clocks are expected to be particularly valuable as they allow for the direct translation of findings in NMRs to humans. Furthermore, our results demonstrate that NMRs age epigenetically despite displaying negligible senescence. This finding underscores that exceptionally long-lived species may evolve mechanisms to uncouple epigenetic aging from physiological decline. We have also identified genes and cellular pathways that impinge on developmental and metabolic processes that are potentially involved in NMR and human longevity. Finally, we demonstrate that NMR queens age more slowly than nonbreeders and identify several genes, including LHX3 transcription factor, involved in pituitary development to be associated with queens’ longevity.

## Methods

### Ethics.

This research complied with all relevant ethical regulations, overseen by four ethics review boards: the University of Rochester Committee on Animal Resources, with protocol number 2009-054 (NMR) and protocol number 2017-033 (mice); Queen Mary University in accordance with the UK Government Animal Testing and Research Guidance (NMR). The human skin samples were acquired with informed consent before collection, approved by the Oxford Research Ethics Committee in the UK, reference 10/H0605/1. The secondary use of the other de-identified/coded human tissue samples (blood, postmortem tissues) is not interpreted as human subjects research under the US Department of Health & Human Services, 45 CFR 46. Therefore, the need to obtain written, informed consent from human study participants was waived (secondary use of de-identified tissues). Human samples were covered by the University of California Los Angeles, Institutional Review Board (IRB) no. 18-000315.

### NMRs.

The NMR tissue samples were provided by two different laboratories: (1) Vera Gorbunova and Andrei Seluanov from the University of Rochester; and (2) Chris Faulkes from the Queen Mary University of London.

#### Animals from the University of Rochester.

NMRs were from the University of Rochester colonies. All animals in the colonies are microchipped and their ages are recorded. Housing conditions are as described previously^41^. All tissues, except for skin biopsies and blood, were obtained from frozen tissue collections at the University of Rochester from healthy animals that were euthanized for other studies. Skin biopsies (2-mm punch) were collected from the backs of the animals under local anesthesia. Blood samples were collected from the tails. The *n* = 3 induced pluripotent stem cells from NMRs were generated as described in ref. ^23^. As control set for the iPS study, we used *n* = 3 fibroblasts samples from animals aged 1 and 2 yr.

Genomic DNA was extracted using Qiagen DNeasy Blood and Tissue kit and quantified using Nanodrop and Qubit.

#### Study animals from Queen Mary University.

NMRs were maintained in the Biological Services Unit at Queen Mary University of London in accordance with the UK Government Animal Testing and Research Guidance. The tissues used in this study were obtained from postmortem specimens from animals free from disease in compliance with national (Home Office) and institutional procedures and guidelines. Because sample collection was from postmortem material, additional local ethical approval was not required for this study. Tissue samples were snap-frozen in liquid nitrogen following dissection and transferred for storage at −80 °C.

### Other species.

The data from other species are presented in other publications^42^ (vervet monkey, ref. ^24^; mice, ref. ^43^). Details on the *n* = 1,211 human tissue samples (adipose, blood, bone marrow, dermis, epidermis, heart, keratinocytes, fibroblasts, kidney, liver, lung, lymph node, muscle, pituitary, skin, spleen) can be found in refs. ^24,44^. Human postmortem tissue and organ samples were obtained from the National NeuroAIDS Tissue Consortium^45^. Additional blood samples were from the Parkinson Environment Gene study and the Cape Town Adolescent Antiretroviral Cohort Study^46^. Skin and other primary cells were provided by Kenneth Raj^47^.

Details on the *n* = 48 liver samples from vervet monkey data (whose ages ranged between zero and 21.8 yr) can be found in ref. ^24^.

The mouse data (strain C57B/l6J) were derived from three tissues: *n* = 111 liver samples (age ranged from 0.08 to 2.25 yr), *n* = 58 blood samples (same age range) and *n* = 42 skin samples (age ranged from 0.25 to 2.5 yr)^43^.

### Methylation array.

The mammalian DNA methylation arrays were profiled using a custom Infinium methylation array (HorvathMammalMethylChip40) based on 37,492 CpG sites^48^. Not all CpGs map to a given species^48^. The genome coordinates for each CpG probe can be found at the GitHub page of the Mammalian Methylation Consortium (Data availability). The manifest file (which includes oligonucleotide sequences and genome coordinates) has been posted on the Gene Expression Omnibus, platform GPL28271. The SeSaMe normalization method was used to define beta values for each probe^49^.

### Statistical analyses.

All reported *P* values are two-sided.

#### Penalized regression models.

Details on the clocks (CpGs, genome coordinates) and R software code are provided in Supplementary Note 1 and Supplementary Table 20. Penalized regression models were created with glmnet^50^. We investigated models produced by ‘elastic net’ regression (alpha = 0.5). The optimal penalty parameters in all cases were determined automatically by using a tenfold internal cross-validation (cv.glmnet) on the training set. The alpha value for the elastic net regression was set to 0.5 (midpoint between Ridge and Lasso type regression) and was not optimized for model performance.

We performed a cross-validation scheme for arriving at unbiased estimates of the accuracy of the different DNA methylation-based age-estimators. One type consisted of leaving out a single sample, Leave-One-Out-Cross Validation from the regression, predicting an age for that sample and iterating over all samples. A critical step is the transformation of chronological age (the dependent variable). While no transformation was used for the pure NMR clocks, we did use a log linear transformation of age for the dual-species clocks of chronological age (Supplementary Note 1).

To introduce biological meaning into age estimates of NMRs and humans which have very different lifespans, as well as to overcome the inevitable skewing due to unequal distribution of data points from NMRs and humans across age ranges, relative age estimation was made using the formula: Relative age = Age/maxLifespan, where the maximum lifespans for the two species (human = 122.5 yr and NMR = 37 yr) were chosen from an updated version of the anAge database^25^.

#### EWASs of age.

EWAS was performed in each tissue separately using the R function ‘standardScreeningNumericTrait’ from the ‘WGCNA’ R package^51^. Next, the results were combined across tissues using Stouffer’s meta-analysis method.

#### GO enrichment analysis.

We analyzed gene set enrichments of all genes that are represented on the mammalian array and that map to the NMR using the genomic region of enrichment annotation tool (GREAT)^52^. We used all CpGs that map to the NMR and that are located on the mammalian array as the background set. The background probes were limited to 16,801 probes that were mapped to the same gene in the NMR genome. Thus, our GREAT enrichment analysis conditioned out (removed) any bias resulting from restricting the analysis to conserved CpGs on the mammalian array platform.

The GREAT software performs both a binomial test (over genomic regions) and a hypergeometric test over genes when using a whole genome background.

We performed the enrichment based on default settings (proximal: 5.0 kilobases (kb) upstream, 1.0 kb downstream; plus distal: up to 1,000 kb) for gene sets associated with GO terms, MSigDB, PANTHER and KEGG pathway. To avoid large numbers of multiple comparisons, we restricted the analysis to the gene sets with between 10 and 3,000 genes. We report nominal *P* values and two adjustments for multiple comparisons: Bonferroni correction and the Benjamini–Hochberg FDR.

## Data availability

The methylation data from the NMRs are available from the Gene Expression Omnibus (GSE174777 and GSE174778). The transcriptomic and proteomic datasets used in the aging study of NMRs and guinea pigs^18^ can be downloaded from GEO (GSE98744). The gene expression data of the NMR queen study^27^ can be downloaded from GEO (GSE98663). The human methylation data, vervet monkey data and mouse data were not generated for this study. These data will be presented in other publications (humans and primates^42^, vervet monkey^24^, mice^43^) and can be requested from S.H. All data from the Mammalian Methylation Consortium will be made publicly available. Genome annotations of these CpGs can be found on GitHub (https://github.com/shorvath/MammalianMethylationConsortium). The mammalian methylation array is available through the nonprofit Epigenetic Clock Development Foundation (https://clockfoundation.org/).

## Extended Data

## Supplementary Material

Supplementary Information

Supplementary Tables

## References

[1] Jarvis JU Eusociality in a mammal: cooperative breeding in naked mole-rat colonies. Science 212, 571–573 (1981). 7209555 10.1126/science.7209555

[2] Buffenstein R The naked mole-rat: a new long-living model for human aging research. J. Gerontol. A Biol. Sci. Med. Sci 60, 1369–1377 (2005). 16339321 10.1093/gerona/60.11.1369

[3] Buffenstein R Negligible senescence in the longest living rodent, the naked mole-rat: insights from a successfully aging species. J. Comp. Physiol. B 178, 439–445 (2008). 18180931 10.1007/s00360-007-0237-5

[4] Lagunas-Rangel FA & Chávez-Valencia V Learning of nature: the curious case of the naked mole rat. Mech. Aging Dev 164, 76–81 (2017). 28472634 10.1016/j.mad.2017.04.010

[5] Lewis KN Unraveling the message: insights into comparative genomics of the naked mole-rat. Mamm. Genome 27, 259–278 (2016). 27364349 10.1007/s00335-016-9648-5 PMC4935753

[6] Taguchi T Naked mole-rats are extremely resistant to post-traumatic osteoarthritis. Aging Cell 19, e13255 (2020). 33112509 10.1111/acel.13255 PMC7681040

[7] Grimes KM, Reddy AK, Lindsey ML & Buffenstein R And the beat goes on: maintained cardiovascular function during aging in the longest-lived rodent, the naked mole-rat. Am. J. Physiol. Heart. Circ. Physiol 307, H284–H291 (2014). 24906918 10.1152/ajpheart.00305.2014 PMC4121653

[8] Delaney MA Initial case reports of cancer in naked mole-rats (*Heterocephalus glaber*). Vet. Pathol 53, 691–696 (2016). 26846576 10.1177/0300985816630796

[9] Tian X High-molecular-mass hyaluronan mediates the cancer resistance of the naked mole rat. Nature 499, 346–349 (2013). 23783513 10.1038/nature12234 PMC3720720

[10] Seluanov A Hypersensitivity to contact inhibition provides a clue to cancer resistance of naked mole-rat. Proc. Natl Acad. Sci. USA 106, 19352–19357 (2009). 19858485 10.1073/pnas.0905252106 PMC2780760

[11] Ruby JG, Smith M & Buffenstein R Naked mole-rat mortality rates defy Gompertzian laws by not increasing with age. eLife 7, e31157 (2018). 29364116 10.7554/eLife.31157 PMC5783610

[12] Smith ZD & Meissner A DNA methylation: roles in mammalian development. Nat. Rev. Genet 14, 204–220 (2013). 23400093 10.1038/nrg3354

[13] Rakyan VK Human aging-associated DNA hypermethylation occurs preferentially at bivalent chromatin domains. Genome Res. 20, 434–439 (2010). 20219945 10.1101/gr.103101.109 PMC2847746

[14] Teschendorff AE Age-dependent DNA methylation of genes that are suppressed in stem cells is a hallmark of cancer. Genome Res. 20, 440–446 (2010). 20219944 10.1101/gr.103606.109 PMC2847747

[15] Lowe R Aging-associated DNA methylation dynamics are a molecular readout of lifespan variation among mammalian species. Genome Biol. 19, 22 (2018). 29452591 10.1186/s13059-018-1397-1 PMC5815211

[16] Horvath S DNA methylation age of human tissues and cell types. Genome Biol. 14, R115 (2013). 24138928 10.1186/gb-2013-14-10-r115 PMC4015143

[17] Horvath S & Raj K DNA methylation-based biomarkers and the epigenetic clock theory of aging. Nat. Rev. Genet 10.1038/s41576-018-0004-3 (2018). 29643443

[18] Heinze I Species comparison of liver proteomes reveals links to naked mole-rat longevity and human aging. BMC Biol. 16, 82 (2018). 30068331 10.1186/s12915-018-0547-y PMC6090990

[19] Field AE DNA methylation clocks in aging: categories, causes, and consequences. Mol. Cell 71, 882–895 (2018). 30241605 10.1016/j.molcel.2018.08.008 PMC6520108

[20] Petkovich DA Using DNA methylation profiling to evaluate biological age and longevity interventions. Cell Metab. 25, 954–960.e956 (2017). 28380383 10.1016/j.cmet.2017.03.016 PMC5578459

[21] Bell CG DNA methylation aging clocks: challenges and recommendations. Genome Biol. 20, 249 (2019). 31767039 10.1186/s13059-019-1824-y PMC6876109

[22] Lowe R DNA methylation clocks as a predictor for aging and age estimation in naked mole-rats, *Heterocephalus glaber*. Aging 12, 4394–4406 (2020). 32126024 10.18632/aging.102892 PMC7093186

[23] Tan L Naked mole rat cells have a stable epigenome that resists iPSC reprogramming. Stem Cell Reports 9, 1721–1734 (2017). 29107597 10.1016/j.stemcr.2017.10.001 PMC5831052

[24] Jasinska AJ Epigenetic clock and methylation studies in vervet monkeys. GeroScience 10.1007/s11357-021-00466-3 (2021). PMC9135907 34591235

[25] de Magalhaes JP, Costa J & Church GM An analysis of the relationship between metabolism, developmental schedules, and longevity using phylogenetic independent contrasts. J. Gerontol. A Biol. Sci. Med. Sci 62, 149–160 (2007). 17339640 10.1093/gerona/62.2.149 PMC2288695

[26] Tacutu R Human aging genomic resources: new and updated databases. Nucleic Acids Res. 46, D1083–D1090 (2018). 29121237 10.1093/nar/gkx1042 PMC5753192

[27] Sahm A Long-lived rodents reveal signatures of positive selection in genes associated with lifespan. PLoS Genet. 14, e1007272 (2018). 29570707 10.1371/journal.pgen.1007272 PMC5884551

[28] Bens M Naked mole-rat transcriptome signatures of socially suppressed sexual maturation and links of reproduction to aging. BMC Biol. 16, 77 (2018). 30068345 10.1186/s12915-018-0546-z PMC6090939

[29] Mulugeta E Molecular insights into the pathways underlying naked mole-rat eusociality. Preprint at bioRxiv 10.1101/209932 (2017).

[30] Kane AE & Sinclair DA Epigenetic changes during aging and their reprogramming potential. Crit. Rev. Biochem. Mol. Biol 54, 61–83 (2019). 30822165 10.1080/10409238.2019.1570075 PMC6424622

[31] Lee SG Naked mole rat induced pluripotent stem cells and their contribution to interspecific chimera. Stem Cell Rep. 9, 1706–1720 (2017). 10.1016/j.stemcr.2017.09.013 PMC5829328 29107591

[32] Matsuyama M, WuWong DJ, Horvath S & Matsuyama S Epigenetic clock analysis of human fibroblasts in vitro: effects of hypoxia, donor age, and expression of hTERT and SV40 largeT. Aging (Albany NY) 11, 3012–3022 (2019). 31113906 10.18632/aging.101955 PMC6555444

[33] Meer MV, Podolskiy DI, Tyshkovskiy A & Gladyshev VN A whole lifespan mouse multi-tissue DNA methylation clock. eLife 7, e40675 (2018). 30427307 10.7554/eLife.40675 PMC6287945

[34] Skulachev VP Neoteny, prolongation of youth: from naked mole rats to “naked apes” (humans). Physiol. Rev 97, 699–720 (2017). 28202600 10.1152/physrev.00040.2015

[35] Sharma K LIM homeodomain factors Lhx3 and Lhx4 assign subtype identities for motor neurons. Cell 95, 817–828 (1998). 9865699 10.1016/s0092-8674(00)81704-3

[36] Granger A The LIM-homeodomain proteins Isl-1 and Lhx3 act with steroidogenic factor 1 to enhance gonadotrope-specific activity of the gonadotropin-releasing hormone receptor gene promoter. Mol. Endocrinol 20, 2093–2108 (2006). 16613990 10.1210/me.2005-0184

[37] Flurkey K, Papaconstantinou J & Harrison DE The Snell dwarf mutation Pit1dw can increase life span in mice. Mech. Aging Dev 123, 121–130 (2002). 11718806 10.1016/s0047-6374(01)00339-6

[38] Bartke A Prolonged longevity of hypopituitary dwarf mice. Exp. Gerontol 36, 21–28 (2001). 11162909 10.1016/s0531-5565(00)00205-9

[39] van Eijk K Genetic analysis of DNA methylation and gene expression levels in whole blood of healthy human subjects. BMC Genomics 13, 636 (2012). 23157493 10.1186/1471-2164-13-636 PMC3583143

[40] Clark SJ, Lee HJ, Smallwood SA, Kelsey G & Reik W Single-cell epigenomics: powerful new methods for understanding gene regulation and cell identity. Genome Biol. 17, 72 (2016). 27091476 10.1186/s13059-016-0944-x PMC4834828

[41] Ke Z, Vaidya A, Ascher J, Seluanov A & Gorbunova V Novel husbandry techniques support survival of naked mole rat (*Heterocephalus glaber*) pups. J. Am. Assoc. Lab. Anim. Sci 53, 89–91 (2014). 24411785 PMC3894653

[42] Horvath S Pan-primate DNA methylation clocks. Preprint at bioRxiv 10.1101/2020.11.29.402891 (2021).

[43] Horvath S Epigenetic clock and methylation studies in gray short-tailed opossums. Preprint at bioRxiv 10.1101/2021.10.13.464301 (2021).

[44] Horvath S Epigenetic clock and methylation studies in the rhesus macaque. GeroScience 10.1007/s11357-021-00429-8 (2021). PMC8599607 34487267

[45] Morgello S The National NeuroAIDS Tissue Consortium: a new paradigm in brain banking with an emphasis on infectious disease. Neuropathol. Appl. Neurobiol 27, 326–335 (2001). 11532163 10.1046/j.0305-1846.2001.00334.x

[46] Horvath S Perinatally acquired HIV infection accelerates epigenetic aging in South African adolescents. AIDS (London, England) 32, 1465–1474 (2018). 10.1097/QAD.0000000000001854 PMC6026068 29746298

[47] Horvath S Epigenetic clock for skin and blood cells applied to Hutchinson Gilford Progeria Syndrome and ex vivo studies. Aging (Albany NY) 10, 1758–1775 (2018). 30048243 10.18632/aging.101508 PMC6075434

[48] Arneson A A mammalian methylation array for profiling methylation levels at conserved sequences. Preprint at bioRxiv 10.1101/2021.01.07.425637 (2021). PMC8831611 35145108

[49] Zhou W, Triche TJ Jr, Laird PW & Shen H SeSAMe: reducing artifactual detection of DNA methylation by Infinium BeadChips in genomic deletions. Nucleic Acids Res. 46, e123–e123 (2018). 30085201 10.1093/nar/gky691 PMC6237738

[50] Friedman J, Hastie T & Tibshirani R Regularization paths for generalized linear models via coordinate descent. J. Stat. Softw 33, 1–22 (2010). 20808728 PMC2929880

[51] Langfelder P & Horvath S WGCNA: an R package for weighted correlation network analysis. BMC Bioinf. 9, 559 (2008). 10.1186/1471-2105-9-559 PMC2631488 19114008

[52] McLean CY GREAT improves functional interpretation of *cis*-regulatory regions. Nat. Biotechnol 10.1038/nbt.1630 (2010). PMC4840234 20436461

