# Supplementary material for: DNA methylation clocks tick in naked mole rats but queens age more slowly than nonbreeders
Source: Nat Aging. Author manuscript; Available in PMC 2022 Apr 1. (PMC8975251; doi:10.1038/s43587-021-00152-1)
Supplement: Supplementary Information [file NIHMS1784575-supplement-Supplementary_Information.pdf]

nature portfolio

https://doi.org/10.1038/s43587-021-00152-1

Supplementary information

DNA methylation clocks tick in naked mole rats but queens age more slowly than nonbreeders

In the format provided by the authors and unedited

nature portfolio

https://doi.org/10.1038/s43587-021-00152-1

Supplementary information

DNA methylation clocks tick in naked mole rats but queens age more slowly than nonbreeders

In the format provided by the authors and unedited

Supplementary Note 1: Technical Details surrounding the DNAm age estimators

Statistical methods used for building the clocks

The epigenetic clocks were used by employing a single elastic net regression model analysis (R function glmnet). We use used Leave-one-out analysis (LOO) using a single lambda value. We chose the following parameters for the glmnet R function (Alpha: 0.5, CV Fold: 10, Lambda choice for Clock: 1 standard error above minimum CV-MSE).

Covariates and coefficient values of the NMR clocks

The clock CpGs, their coefficient values, and genomic locations are presented in **Supplemental Table S20**.

1) Version 1 of the NMR pan tissue clock is based on 47 CpGs whose coefficient values are specified in the column "NMR.PanTissueVersion1" in **Supplemental Table S20**. Age transformation=identity, i.e. F(Age)=Age. This clock was described in our main article.
2) Version 2 of the NMR pan tissue clock is based on 191 CpGs whose coefficient values are specified in the column "NMR.PanTissueVersion2". Age transformation=identity, i.e. F(Age)=Age. This clock used our iPSC data in the training set.
3) Tissue specific clocks for NMR have been created for blood (40 CpGs specified in Coef.NMR.Blood), skin (36 CpGs in Coef.NMR.Skin), liver (27 CpGs in Coef.NMR.Liver), kidney (26 CpGs in Coef.NMR.Kidney).
4) The human-NMR clock for chronological age is based on 498 CpGs whose coefficient values are specified in the column "Coef.HumanNMRLogLinearAge". Age transformation=log-linear described below.
5) The human NMR clock for relative age is based on 509 CpGs whose coefficient values are specified in the column "HumanNMR.RelativeAge". Age transformation: relative age. i.e. F(Age)=Age/maxLifespan. Max lifespan for NMRs is 37 years. Human max lifespan =122.5 years.

General description of age transformation

The human-NMR clocks for chronological age used log linear transformations that are similar to those employed for the HUMAN pan tissue (Horvath 2013) [1].

An elastic net regression model (implemented in the glmnet R function) was used to regress a transformed version of age on the beta values in the training data. The glmnet function requires the user to specify two parameters (alpha and beta). Since I used an elastic net predictor, alpha was set to 0.5. But the lambda value of was chosen by applying a 10 fold cross validation to the training data (via the R function cv.glmnet).

The elastic net regression results in a linear regression model whose coefficients b_0, b_1, . . . , relate to transformed age as follows

$F(\text{chronological age})=b_0+b_1 CpG_1+ \ldots +b_p CpG_p+\text{error}$

Note that the intercept term is denoted by b_0. The coefficient values can be found in the attached Excel file.

Based, on the coefficient values from the regression model, DNAmAge is estimated as follows
*DNAm*Age=F^{-1}(b_0+$b_1 CpG_1$+ . . . +$b_p CpG_p$)

where $F^{-1}(y)$ denotes the mathematical inverse of the function F(.). Thus, the regression model can be used to predict to transformed age value by simply plugging the beta values of the selected CpGs into the formula.

Defining Properties of the log linear transformation

As indicated by its name, the "log-linear" function, has a logarithmic dependence on age before the average age of sexual maturity (of the species) and a linear dependence after Age at Sexual Maturity (of the species). For the human-NMR clocks we used the following averages at sexual maturity (in units of years): 13.5 years for humans and 5 years for NMRs.
The life history traits come from the data base anAge.

Construction

We used a piecewise transformation, parameterized by Age of Sexual Maturity (A).
The transformation is F(x), given by

$$F(x) = g\left(\frac{x+1.5}{A+1.5}\right) \text{ where } g(t) = \begin{cases} \log(t), & for\ 0 \le t \le 1 \\ t-1, & for\ 1 \le t \end{cases}$$

Explicitly, F(x) is given by

$$F(x) = \begin{cases} \log\left(\frac{x+1.5}{A+1.5}\right), & for\ 0 \le x \le A \\ \frac{x-A}{A+1.5}, & for\ A \le x \end{cases}$$

In order to use this transformation to predict Age on ***new samples***, one needs to use the *inverse* transformation, $F^{-1}(y)$, given by

$$F^{-1}(y) = \begin{cases} (A+1.5) * \exp(y) - 1.5, & for\ y \le 0 \\ (A+1.5)y + A, & for\ y \ge 0 \end{cases}$$

For predicting age, apply the inverse transformation to coefficient-weighted sum. That is,

$$DNAmAge = F^{-1}(x * \beta)$$

where β is the vector of coefficients and x is the vector of methylation values, with an intercept term.

The DNAm Age estimate is estimated in two steps.

First, one forms a weighted linear combination of the CpGs whose details can be found in the supplementary Excel file (Table S8)

The file reports the probe identifier (cg number) used in the custom Infinium array (HorvathMammalMethylChip40). The weights used in this linear combination are specified in the respective column entitled "Coef.".
The formula assumes that the DNA methylation data measure "beta" values but the formula could be adapted to other ways of generating DNA methylation data.

R Implementation of the log linear transformation

```
### Applies the log linear transformation to the input vector x,i.e. to Age
F= Vectorize(function(x, maturity, ...) {
  if (is.na(x) | is.na(maturity)) {return(NA)}
  k <- 1.5
  y <- 0
  if (x < maturity) {y = log((x+k)/(maturity+k))}
  else {y = (x-maturity)/(maturity+k)}
  return(y)
})
### Inverse log linear trnasformation
F.inverse= Vectorize(function(y, maturity, ...) {
  if (is.na(y) | is.na(maturity)) {return(NA)}
  k <- 1.5
  x <- 0
  if (y < 0) {x = (maturity+k)*exp(y)-k}
  else {x = (maturity+k)*y+maturity}
  return(x)
})
```

```
# R function for multivariate regression model
multivariatePredictorCoef=function(dat0, datCOEF,imputeValues=FALSE) {
datout=data.frame(matrix(NA,nrow=dim(dat0)[[2]]-1,ncol=dim(datCOEF)[[2]]-1 ))
match1=match(datCOEF[-1,1],dat0[,1] )
if (   sum(!is.na(match1))==0 ) stop("Input error. The first column of dat0 does not contain CpG
identifiers (cg numbers).")
dat1=dat0[match1,]
row.names1=as.character(dat1[,1])
dat1=dat1[,-1]
if (imputeValues ){dat1=impute.knn(data=as.matrix(dat1) ,k = 10)[[1]]}
for (i in 1:dim(dat1)[[2]] ){ for (j in 2:dim(as.matrix(datCOEF))[[2]] ){
datout[i,j-1]=sum(dat1[,i]* datCOEF[-1,j],na.rm=TRUE)+ datCOEF[1,j]}}
colnames(datout)=colnames(datCOEF)[-1]
rownames(datout)=colnames(dat0)[-1]
datout=data.frame(SampleID= colnames(dat0)[-1],datout)
datout
} # end of function

datCoef=read.csv("TableS8.csv")
names(datCoef)
```

The first columns should read as follows

```
var
```

```
Coef.NMR.PanTissueVersion1
Coef.NMR.PanTissueVersion2
Coef.NMR.Blood
Coef.NMR.Skin
Coef.NMR.Liver
Coef.NMR.Kidney
Coef.HumanNMR.AgeLogLinear
Coef.HumanNMR.RelativeAge
```

```
# Restrict attention to the first 8 columns
datCoef=datCoef[,c(1:8)]

match1=match(datCoef[-1,1],dat0[,1] )
missingProbes= as.character(datCoef[-1,1] )[is.na(match1)]

dat1=dat0[match1,]
# data frame with predicted values.
datPredictions=multivariatePredictorCoef(dat1,datCOEF=datCoef,imputeValues=FALSE)

#let's relabel the columns by replacing "Coef" with "DNAm" since the columns contain estimates of age or relative age instead of coefficient values
colnames(datPredictions)=gsub(pattern="Coef", replacement="DNAm", x=colnames(datPredictions))

# We need to transform the human NMR clock for chronological age using the inverse of the log linear transformation.
datPredictions$DNAm.HumanNMR.AgeLogLinear=
F.inverse(datPredictions$DNAm.HumanNMR.AgeLogLinear, maturity=5)
```

The data frame "datPredictions" contains the age estimates in units of years and relative age estimates.

References

1 Horvath, S. DNA methylation age of human tissues and cell types. *Genome Biol* **14**, R115, doi:10.1186/gb-2013-14-10-r115 (2013).
